# Exome sequencing in multiple sclerosis families identifies 12 candidate genes and nominates biological pathways for the genesis of disease

**DOI:** 10.1371/journal.pgen.1008180

**Published:** 2019-06-06

**Authors:** Carles Vilariño-Güell, Alexander Zimprich, Filippo Martinelli-Boneschi, Bruno Herculano, Zhe Wang, Fuencisla Matesanz, Elena Urcelay, Koen Vandenbroeck, Laura Leyva, Denis Gris, Charbel Massaad, Jacqueline A. Quandt, Anthony L. Traboulsee, Mary Encarnacion, Cecily Q. Bernales, Jordan Follett, Irene M. Yee, Maria G. Criscuoli, Angela Deutschländer, Eva M. Reinthaler, Tobias Zrzavy, Elisabetta Mascia, Andrea Zauli, Federica Esposito, Antonio Alcina, Guillermo Izquierdo, Laura Espino-Paisán, Jorge Mena, Alfredo Antigüedad, Patricia Urbaneja-Romero, Jesús Ortega-Pinazo, Weihong Song, A. Dessa Sadovnick

**Affiliations:** 1 Department of Medical Genetics, University of British Columbia, Vancouver, Canada; 2 Department of Neurology, Medical University of Vienna, Vienna, Austria; 3 Laboratory of Human Genetics of Neurological Disorders, CNS Inflammatory Unit, Institute of Experimental Neurology, IRCCS San Raffaele Scientific Institute, Milan, Italy; 4 MS Unit and Department of Neurology, IRCCS Policlinico San Donato, Milan, Italy; 5 Department of Biomedical Sciences for Health, University of Milan, Milan, Italy; 6 Townsend Family Laboratories, Department of Psychiatry, University of British Columbia, Vancouver, Canada; 7 National Clinical Research Center for Geriatric Diseases, Xuanwu Hospital of the Capital Medical University, Beijing, China; 8 Department of Cell Biology and Immunology, Instituto de Parasitología y Biomedicina López Neyra (IPBLN), CSIC, Granada, Spain; 9 Immunology Dept, Hospital Clínico San Carlos, Instituto de Investigación Sanitaria del Hospital Clínico San Carlos (IdISSC), Madrid, Spain; 10 Red Española de Esclerosis Múltiple REEM, Madrid, Spain; 11 Achucarro Basque Center for Neuroscience, Universidad del País Vasco (UPV/EHU), Leioa, Spain; 12 Ikerbasque, Basque Foundation for Science, Bilbao, Spain; 13 Instituto de Investigación Biomédica de Málaga-IBIMA, Unidad de Gestion Clínica de Neurociencias, Hospital Regional Universitario de Málaga, Málaga, Spain; 14 Division of Immunology, Department of Pediatrics, CR-CHUS, Faculty of Medicine and Health Sciences, University of Sherbrooke, Sherbrooke, Canada; 15 Toxicology, Pharmacology and Cell Signalisation—UMR-S 1124 Université Paris Descartes, Paris, France; 16 Department of Pathology, Faculty of Medicine, University of British Columbia, Vancouver, Canada; 17 Division of Neurology, Faculty of Medicine, University of British Columbia, Vancouver, Canada; 18 Department of Neurology, Mayo Clinic Florida, Jacksonville, FL, United States of America; 19 Department of Clinical Genomics, Mayo Clinic Florida, Jacksonville, FL, United States of America; 20 Department of Neuroscience, Mayo Clinic Florida, Jacksonville, FL, United States of America; 21 Unidad de Esclerosis Múltiple, Hospital Virgen Macarena, Sevilla, Spain; 22 Neurology Department, Hospital Universitario de Cruces, S/N, Baracaldo, Spain; University of Pennsylvania, UNITED STATES

## Abstract

Multiple sclerosis (MS) is an inflammatory disease of the central nervous system characterized by myelin loss and neuronal dysfunction. Although the majority of patients do not present familial aggregation, Mendelian forms have been described. We performed whole-exome sequencing analysis in 132 patients from 34 multi-incident families, which nominated likely pathogenic variants for MS in 12 genes of the innate immune system that regulate the transcription and activation of inflammatory mediators. Rare missense or nonsense variants were identified in genes of the fibrinolysis and complement pathways (*PLAU*, *MASP1*, *C2*), inflammasome assembly (*NLRP12*), Wnt signaling (*UBR2*, *CTNNA3*, *NFATC2*, *RNF213*), nuclear receptor complexes (*NCOA3*), and cation channels and exchangers (*KCNG4*, *SLC24A6*, *SLC8B1*). These genes suggest a disruption of interconnected immunological and pro-inflammatory pathways as the initial event in the pathophysiology of familial MS, and provide the molecular and biological rationale for the chronic inflammation, demyelination and neurodegeneration observed in MS patients.

## Author summary

Although the majority of patients diagnosed with multiple sclerosis do not have a family history of disease, 13% report having a close relative also diagnosed with multiple sclerosis. In these families, the cause of multiple sclerosis can be largely attributed to a single genetic variant that is transmitted through generations. In this study we analyzed DNA from 132 patients from 34 families, resulting in the identification of 12 rare genetic variants that are largely responsible for the onset of multiple sclerosis in these families. These variants are located in genes implicated in specific immunological pathways, and suggest the biological mechanisms that trigger the onset of multiple sclerosis. These genes and variants provide the means for the generation of cellular and animal models of human disease, and highlight biological targets for the development of novel treatments.

## Introduction

Multiple sclerosis (MS) is a common autoimmune disease of the central nervous system (CNS) affecting over two million people worldwide [1]. Although described as early as the 14^th^ century, it was Jean-Martin Charcot in 1868 who recognised MS as a distinct entity, and provided the first detailed description of its clinical and pathological features [2]. Knowledge of the biological processes involved in the onset of MS have advanced greatly, and an increasing number of disease-modifying treatments (DMTs) have been approved since the 1990s; however, a cure has remained elusive [3, 4]. A better understanding of the molecular mechanisms orchestrating the disruption of biological processes in MS patients is critical for the development of efficacious treatments that address the causes of MS and its progression, enhance remyelination, and prevent axonal loss and disability [5]. Large scale genome-wide association studies (GWAS) have already identified more than 200 genes that can moderately affect the individual’s susceptibility to the disease [6]. Given the large size of these case-control studies, risk variants that remain undiscovered to date are expected to be individually rare. Thus, we implemented high-throughput second generation sequencing technologies in multi-incident MS families for the identification of rare disease-causing variants. Although the majority of patients do not present a family history of MS, the prevalence of familial aggregation has been estimated at 12.6% globally [7]. In these families, rare variants co-segregating with MS are likely to account for the highest attributable risk towards the disease; however, additional genetic and environmental factors are expected to play a significant role in the presentation of clinical symptoms, level of disability, disease progression, penetrance and onset age [8, 9]. The application of whole-exome sequencing (WES) in MS families has already nominated pathogenic mutations in *NR1H3*, *P2RX4/P2RX7*, *NLRP1* and *GALR2* [9–12]. Although only one of these discoveries has been replicated [13], mutations responsible for Mendelian forms of MS highlight the molecular mechanisms underlying the cause of disease, and provide the means for the generation of new cellular and animal models of MS based on human genetic etiology [9]. The comprehensive characterization of the biological pathways disrupted in these models will nominate targets for pharmaceutical intervention trials and precision medicine approaches. In addition, genetic screening for these pathogenic variants will enable the identification of at risk individuals, provide confirmation of diagnosis, and facilitate the prediction of disease prognosis and treatment efficacy [14, 15]. This is critical to improve quality of life for MS patients, as early diagnosis and selection of effective DMTs have been associated with improved patient outcomes, and reduced accumulation of irreversible neurological damage [16].

## Results

**Fig 1:**
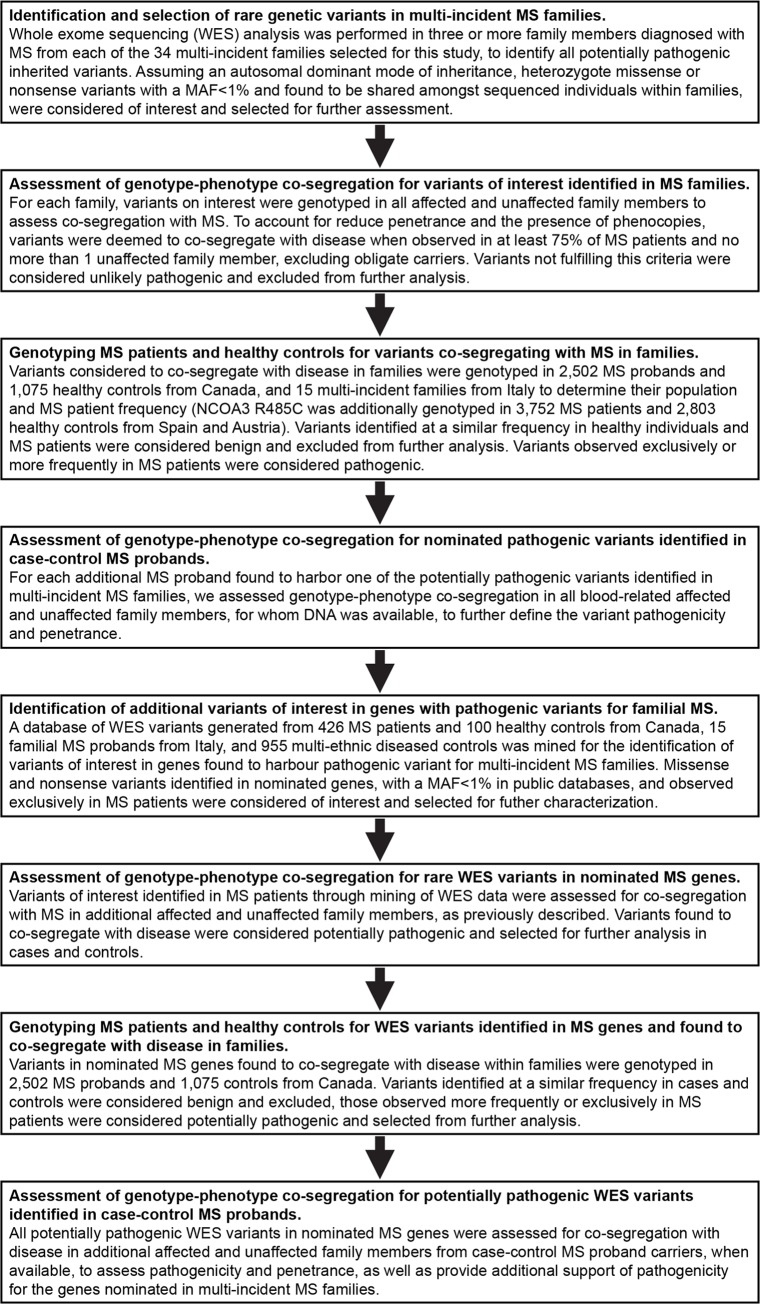
Flowchart describing the methodology and samples characterized in this study.

**Fig 2:**
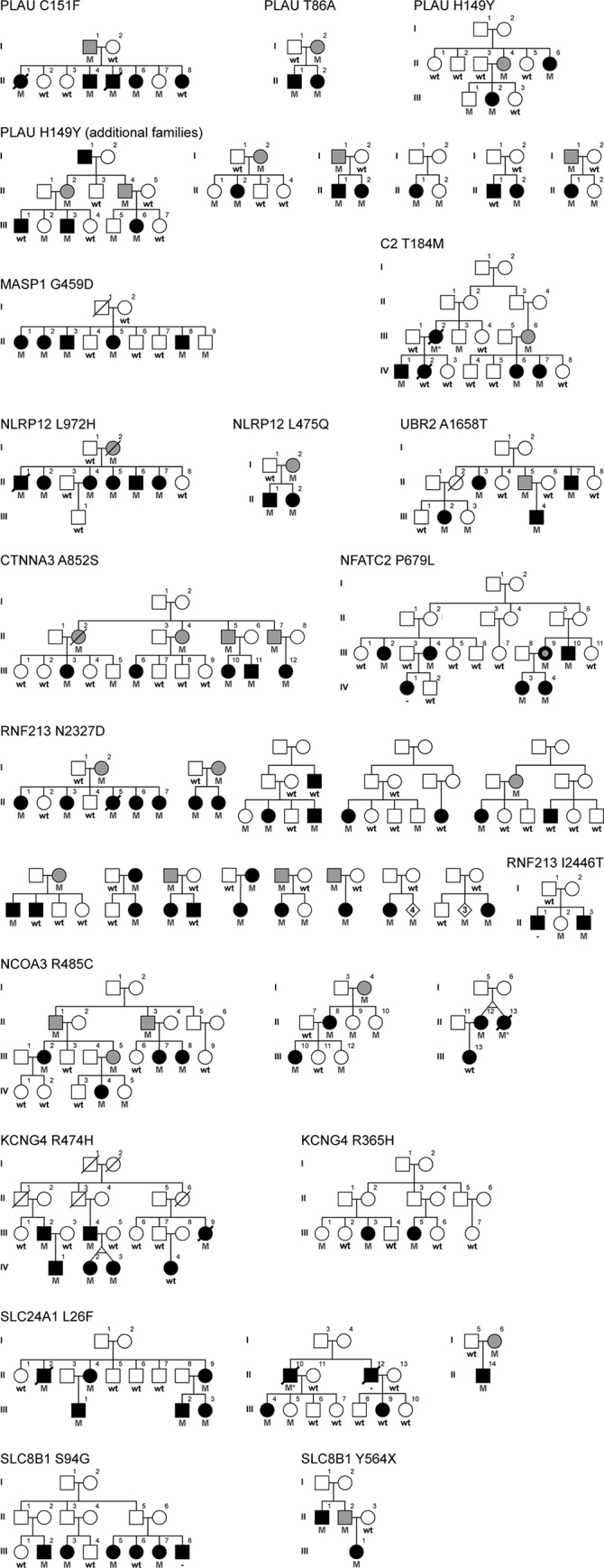
Pedigrees for families nominating genes for MS. Males are represented by squares and females by circles, a diagonal line indicates subjects known to be deceased. Black filled symbol, MS; black filled with gray dot, possible MS; gray filled, unaffected obligate carrier. Heterozygote carriers (M) and wild-type (wt) genotypes are provided. MS patients with inferred genotypes are indicated with an asterisk.

**Fig 3:**
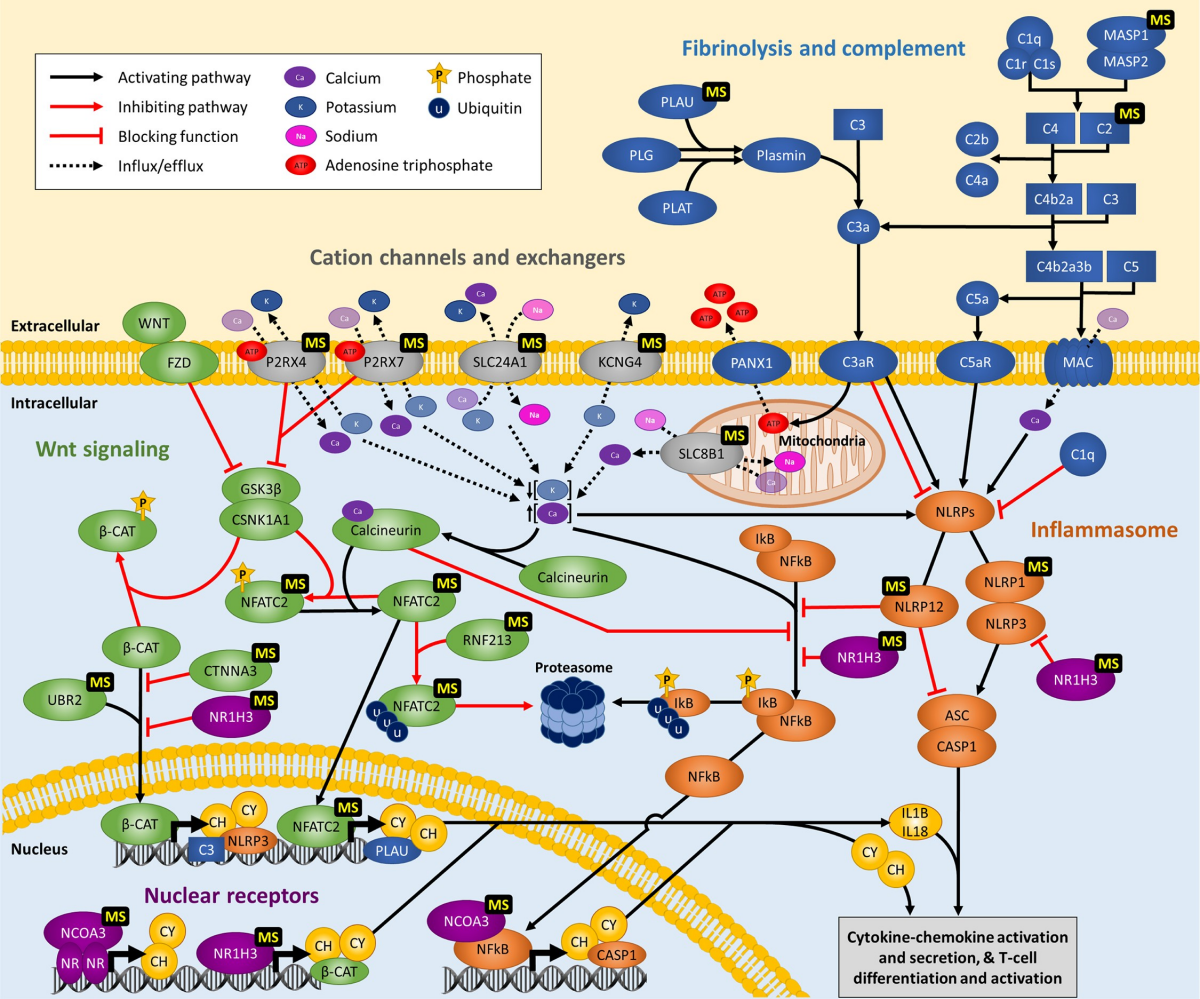
Pathways predicted to be disrupted in multi-incident MS families. Simplified representation of immunological pathways containing genes harboring disease-causing variants identified in MS patients, highlighting their crosstalk and overlap. Genes of the fibrinolysis and complement cascades are provided in blue, inflammasome activation in orange, Wnt signaling pathways in green, nuclear receptor complexes in purple, and cation channels and exchangers in gray. Genes harboring variants segregating with MS in families are indicated with a ‘MS’ label. NR, nuclear receptor; CH, chemokine; CY, cytokine.

A flowchart describing the samples and methodology implemented in this study is provided in Fig 1. To identify genetic variants of major effect responsible for Mendelian forms of MS, we performed WES analysis in 132 MS patients from 34 multi-incident families of European descent. The high incidence of MS observed in these families, together with a high ratio of MS patients to healthy siblings (>25%), lack of consanguinity or gender bias, and the presence of unaffected parents, suggest autosomal dominant with reduced penetrance as the most plausible disease model. Thus we evaluated pathogenicity for all heterozygote missense or nonsense variants on autosomes with a minor allele frequency (MAF) below 1% in private or public databases of variants [17], by assessing co-segregation with MS. To account for phenocopies and reduced penetrance, variants were considered to segregate with disease when observed in at least 75% of blood-related individuals diagnosed with MS, and no more than one unaffected family member, excluding unaffected parents of MS patients; defined as obligate carriers. Using this a priory criterion, the implementation of WES identified disease-causing variants co-segregating with MS in 12 families (Fig 2). Rare missense or nonsense variants were identified in plasminogen activator, urokinase (PLAU p.Cys151Phe), mannan binding lectin serine peptidase 1 (MASP1 p.Gly459Asp), complement component 2 (C2 p.Thr184Met), NLR family pyrin domain containing 12 (NLRP12 p.Leu972His), ubiquitin protein ligase E3 component N-recognin 2 (UBR2 p.Ala1658Thr), catenin alpha 3 (CTNNA3 p.Ala852Ser), nuclear factor of activated T-cells 2 (NFATC2 p.Pro679Leu), ring finger protein 213 (RNF213 p.Asn2327Asp), nuclear receptor coactivator 3 (NCOA3 p.Arg485Cys), potassium voltage-gated channel modifier subfamily G member 4 (KCNG4 p.Arg474His), solute carrier family 24 member 1 (SLC24A1 p.Leu26Phe), and solute carrier family 8 member B1 (SLC8B1 p.Ser94Gly) (Table 1). Interestingly, these genes appear to cluster within linked immunological pathways, suggesting a common biological process underlying the onset of MS in families (Fig 3), and provide a molecular and biological rationale for the chronic inflammation, demyelination and neurodegeneration observed in MS patients.

**Table 1:** Genetic variants of interest or considered to co-segregate with MS in multi-incident families. Genomic coordinates from NCBI Build 37.1 (hg19) and dbSNP refSNP (rs) identifiers from build 150 or submitted SNP (ss) numbers are provided. Sample counts and/or minor allele frequency (MAF) for MS patients, healthy controls and the Exome Aggregation Consortium (ExAC) database are given. Estimated effect on protein function was assessed with the Combined Annotation Dependent Depletion (CADD) phred-scale scores v1.4. n/a, not available.

Gene	Genomic position	Reference/ Alternative	cDNA change	Amino acid change	dbSNP ID rs/ss	MS patients n (MAF)	Controls n (MAF)	ExAC MAF	CADD
PLAU	10:75672744	A/G	c.256A>G	p.T86A	rs753886853	1 (0.02%)	0	0.004%	7.1
	10:75673124	C/T	c.445C>T	p.H149Y	rs117135013	7 (0.14%)	1 (0.05%)	0.06%	6.7
	10:75673131	G/T	c.452G>T	p.C151F	ss3646580234	1 (0.02%)	0	n/a	24.7
MASP1	3:186954283	C/T	c.1376G>A	p.G459D	ss3646580232	1 (0.02%)	0	n/a	26.2
	3:186954275	G/T	c.1384C>A	p.P462T	ss2137543902	1 (0.02%)	0	n/a	27.1
C2	6:31901495	C/T	c.551C>T	p.T184M	rs138034438	1 (0.02%)	0	0.008%	22.2
NLRP12	19:54313859	G/A	c.1054C>T	p.R352C	rs199881207	1 (0.02%)	1 (0.05%)	0.035%	23.3
	19:54313489	A/T	c.1424T>A	p.L475Q	ss3646580242	1 (0.02%)	0	n/a	23.6
	19:54301509	A/T	c.2915T>A	p.L972H	ss3646580241	1 (0.02%)	0	n/a	24.9
UBR2	6:42656072	G/A	c.4972G>A	p.A1658T	ss3646580233	1 (0.02%)	0	n/a	32.0
CTNNA3	10:67680222	C/A	c.2554G>T	p.A852S	rs778712224	1 (0.02%)	0	0.001%	26.2
NFATC2	20:50049290	G/A	c.2036C>T	p.P679L	ss3646580244	1 (0.02%)	0	n/a	27.1
RNF213	17:78319114	A/G	c.6979A>G	p.N2327D	rs138044665	13 (0.26%)	0	0.10%	4.3
	17:78319472	T/C	c.7337T>C	p.I2446T	ss3646580239	1 (0.02%)	0	n/a	16.5
	17:78341843	C/T	c.12055C>T	p.R4019C	rs139265462	5 (0.10%)	0	0.05%	15.4
NCOA3	20:46264406	C/T	c.1453C>T	p.R485C	rs138250384	3 (0.06%)	0	0.02%	26.8
KCNG4	16:84256289	C/T	c.1094G>A	p.R365H	rs553198108	1 (0.02%)	0	0.002%	29.0
	16:84255962	C/T	c.1421G>A	p.R474H	rs761759691	1 (0.02%)	0	0.008%	24.0
SLC24A1	15:65916494	C/T	c.76C>T	p.L26F	rs755508009	3 (0.06%)	0	0.007%	15.9
SLC8B1	12:113759030	T/C	c.280A>G	p.S94G	rs754992099	1 (0.02%)	0	0.001%	0.005
	12:113737645	G/C	c.1692C>G	p.Y564X	ss3646580235	1 (0.02%)	0	n/a	39.0

### Fibrinolysis and the complement cascade

Fibrinolysis is the process responsible for dissolving fibrin of blood clots and promote tissue repair and remodeling following vascular lesion. Plasmin is the primary fibrinolysin, and is the active enzyme from the proteolysis of plasminogen (PLG) by serine proteases, plasminogen activator tissue type (PLAT) or PLAU (Fig 3) [18]. Components of the PLG activation system have been found to play a role in cardiovascular diseases, cancer proliferation, and inflammatory diseases, including sepsis, metabolic disease, and arthritis [19]. In MS, a rare genetic variant in PLG (p.Gly420Asp) was found to be over-represented in patients compared to healthy controls [20]. In addition, several neurological diseases, including MS, present abundant CNS deposition of fibrinogen resulting in microglial activation, axonal damage, and inhibition of oligodendrocyte differentiation and remyelination [21, 22]. Interestingly, fibrin has been suggested as a promising therapeutic target for neurological diseases, as its depletion is protective against inflammatory demyelination in animal models [21].

The complement system consists of a large collection of plasma proteins that can be activated in a cascade-like fashion in response to invading pathogens and damaged host cells. Crosstalk between the fibrinolysis and complement systems has been well described, and includes plasmin which is capable of effectively cleaving complement components C3 and C5 into their active forms [23]. The activation of the complement leads to opsonisation of pathogens for phagocytosis, anaphylatoxin production to promote inflammation, and the assembly and deposition of the membrane attack complex which disrupts membrane integrity resulting in the death of targeted bacteria and infected or damaged cells (Fig 3) [24, 25]. The complement system has been linked to MS pathophysiology, with deposition of active complement components within brain plaques, peri-plaques and adjacent white matter regions [26, 27]. In addition, complement components play a role in microglial activation, neuroinflammation, and synaptic loss in neurodegenerative diseases [28, 29].

#### Plasminogen activator, urokinase (PLAU)

The implementation of WES in five siblings diagnosed with MS identified a PLAU p.Cys151Phe substitution in four of the five family members diagnosed with MS (Fig 2). A single rare missense or nonsense variant shared by all affected individuals was not observed. The age at onset of MS for patients harboring PLAU p.Cys151Phe was between the ages of 23 and 36 years (mean = 31.0, standard deviation (SD) ± 7.0). Clinical information for this family is limited as II-1 and II-5 were deceased by 52 and 44 years of age, respectively. The clinical course for PLAU p.Cys151Phe carriers was consistent with relapsing remitting (RR)MS at the onset of disease. II-6 presented a disability score (EDSS, Expanded Disability Status Scale) [30] of 2.0 at MS onset (S1 Table). The PLAU p.Cys151Phe was inherited from the father (I-1), who at 71 years of age did not report suffering from MS symptoms or any neurological disorder. A seemingly healthy female (II-7), who was 34 years old at the most recent interview, was also found to carry the PLAU substitution. We were unable to obtain an update on the disease status for II-7, but given the mean age at onset of MS in this family, we can not rule out that she may have developed MS later in life.

**Fig 4:**
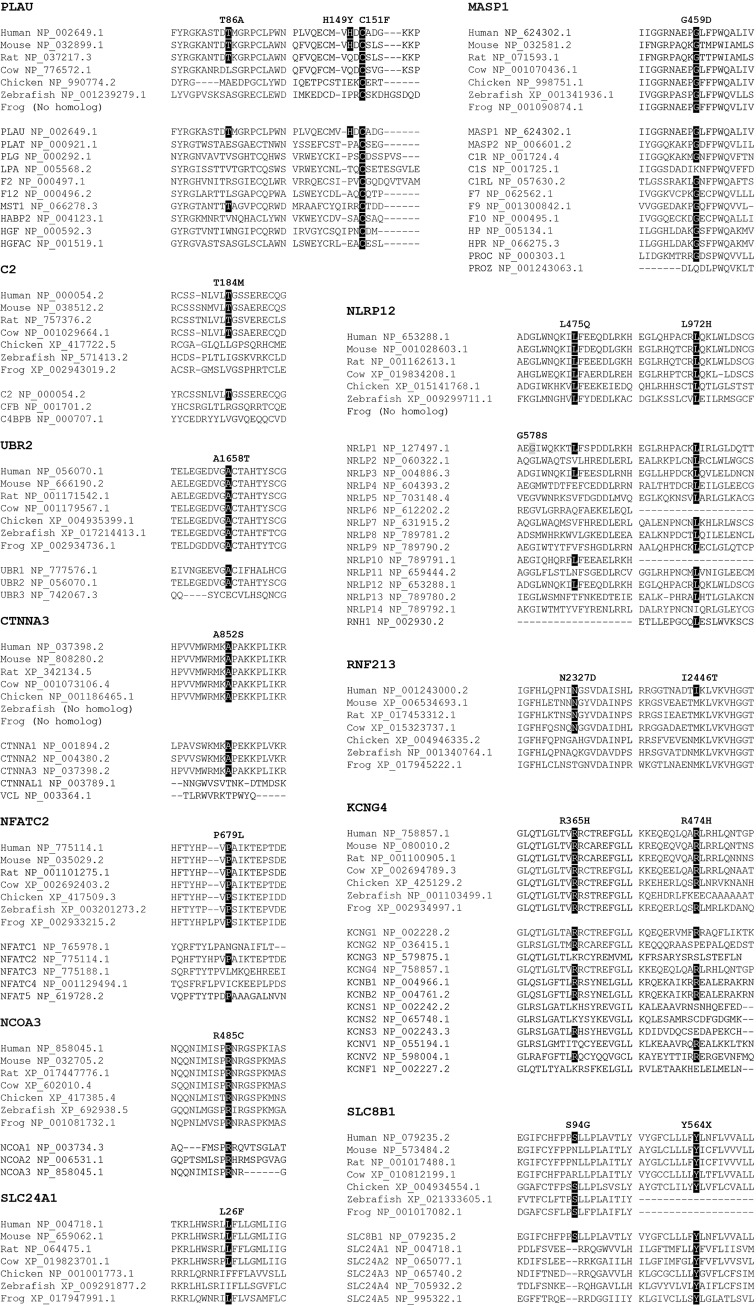
Protein conservation in orthologs and human paralogs. Organism and RefSeq accession numbers are provided for orthologs and gene name and RefSeq accession numbers for human paralogs, which were obtained from Ensembl release 91. Evolutionarily conserved positions for nominated pathogenic variants are highlighted in black, and the NLRP1 p.Gly578Ser mutation previously identified in MS patients indicated in gray [11].

**Fig 5:**
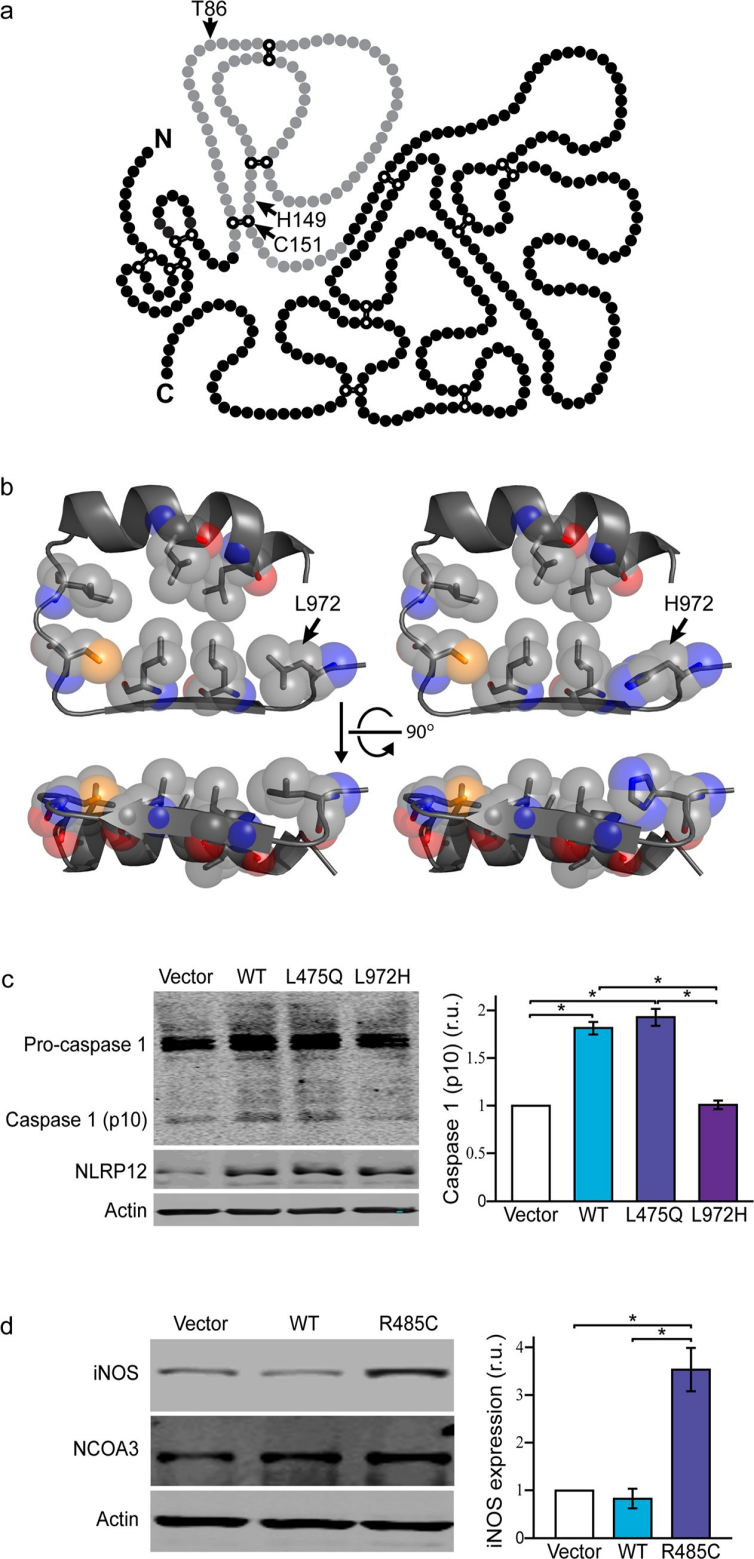
Functional analysis. a) Primary structure of single-chain PLAU protein, adapted from Berkenblit et al. 2005 [32]. Kringle domain is provided in gray, with cysteine residues forming disulfide bonds indicated in white. Amino acid substitutions identified in MS families are shown. b) Crystal structure for NLRP12 leucine rich repeat (LRR) sixth domain showing conserved amino acid residues was predicted from NP_653288.1 with I-TASSER (Iterative Threading ASSEmbly Refinement) [181], and the p.Leu972His substitution introduced using PyMol 1.7. c) Western blot showing expression of pro-caspase-1, caspase-1, NLRP12 and β-actin in microglial (BV2) cells transfected with an empty vector (vector), wild-type NLRP12 (WT) or mutant constructs (L475Q or L972H); and d) expression of iNOS, NCOA3 and β-actin in microglial (BV2) cells transfected with an empty vector (vector), wild-type NCOA3 (WT) or NCOA3 p.Cys485 (R485C). Histograms depict mean expression ± standard error. *Tukey’s HSD post hoc p-value < 0.001. r.u. relative units.

PLAU p.Cys151Phe replaces an evolutionarily conserved cysteine (Fig 4) and is predicted damaging on protein function, with a Combined Annotation Dependent Depletion-phred (CADD) score of 24.7 [31]. The affected residue is one of the six cysteines required for the formation of disulfide bonds that maintain a kringle domain structure (Fig 5A) [32]. Kringle domains are common structures in proteinases associated with blood clotting and the fibrinolysis system, and play a role in the regulation of proteolytic activity. The kringle domain in PLAU is necessary for the interaction with its specific inhibitor SERPINE1, also known as plasminogen activator inhibitor-1 (PAI-1), and prevent the cleavage of PLG into plasmin [18, 33]. The use of a SERPINE1 antagonist in the experimental autoimmune encephalomyelitis (EAE) animal model of MS was shown to decrease inflammation, demyelination, and axonal degeneration. In contrast, induction of EAE in PLAT knock out mice, which have impaired fibrin degradation, causes exacerbated disease and delayed recovery [34, 35]. Taken together, these studies support a role for fibrinolysis and PLAU p.Cys151Phe in the pathophysiology of MS.

The PLAU substitution identified in this family has not been previously described, and genotyping p.Cys151Phe in 2,502 MS probands and 1,075 healthy controls from Canada, and 15 multi-incident families from Italy did not identify any additional carriers. Mining WES data from 426 MS patients from Canada led to the identification of two missense variants (p.Thr86Ala, p.His149Tyr) with a MAF below 1% in public databases [17], and not present in our WES control samples, which consist of 100 healthy individuals from Canada and 955 multi-ethnic diseased controls. Genotyping of PLAU p.Thr86Ala in additional family members identified this rare variant in the proband’s sibling (II-1), who was diagnosed with MS at 27 years of age, and their mother (I-2) for whom no information is available (Fig 2). PLAU p.His149Tyr was observed in one additional family member diagnosed with MS (III-2), an unaffected brother (III-1) who was 26 years old at interview, and their mother (II-4) who at 62 years of age did not disclose suffering from MS symptoms. These two variants fulfil our criteria for co-segregation with disease, and were genotyped in MS probands and controls from Canada. PLAU p.Thr86Ala was not observed in any additional samples, in contrast p.His149Tyr was identified in six additional MS probands and one healthy control (Table 1). Genotyping p.His149Tyr in family members identified the variant in eight out of ten MS patients for whom DNA was available, but also five unaffected family members and five obligate carriers (Fig 2). Although most MS patients in these families presented the p.His149Tyr variant, several families do not support our criteria for co-segregation with disease.

WES analysis of 15 familial probands from Italy identified one additional variant in PLAU, (p.Ser138Thr) not co-segregating with disease in two families (S2 Table), and the previously described p.His149Tyr substitution in one MS patient; however this variant was not present in one additional family member diagnosed with MS. Although p.His149Tyr was not found to co-segregate with MS in several families, its close physical proximity to p.Cys146 and p.Cys151, two amino acid residues forming disulfide bonds in the kringle structure (Fig 5A); together with an elevated frequency in Canadian MS patients (MAF = 0.14%) compared to controls (MAF = 0.05%), resulting in an odds ratio (OR) of 2.85 (95% confidence interval (CI) = 0.35–23.25), and the identification of this rare missense variant in multi-incident families from Canada and Italy, suggest that p.His149Tyr may represent a risk factor for MS. This is further supported by a significant association (*p* = 4.7×10^−8^) between common genetic variants in PLAU and MS susceptibility risk reported in the largest GWAS of MS to date [6]. PLAU is also a plausible biological candidate for a role in MS, as the level of PLAU expression in circulating monocytes has been shown to correlate with clinical activity in RRMS patients, and to be permanently elevated in patients with secondary progressive (SP)MS. Similarly, a correlation between increased PLAU expression and disease severity score in MS patients has also been described [36].

#### Mannan binding lectin serine peptidase 1 (MASP1)

Exome analysis of five siblings diagnosed with MS from a large multi-incident German family identified a rare variant in MASP1 p.Gly459Asp segregating with disease (Fig 2). This substitution was identified in all family members diagnosed with MS, and one healthy male sibling (II-9) who was 44 years of age at examination. Affected MASP1 p.Gly459Asp carriers presented a clinical course consistent with RRMS at the onset of disease, which became progressive for II-1, II-5 and II-8. The average age at the onset of MS was 27.4 years (SD ± 6.3) with disease severity that despite long disease duration does not appear to be highly disabling for most family members (S1 Table). The majority of MS patients in this family had depression, which was severe for II-1 and II-2, and accompanied by hallucinations and delusions in II-5.

Genotyping MASP1 p.Gly459Asp in MS probands and healthy controls from Canada, and Italian families did not identify any additional carriers (Table 1). Mining WES data identified three additional rare MASP1 variants in MS patients not observed in controls (p.Arg441His, p.Pro462Thr, p.Arg538Ter). Genotyping these variants within each family identified less than 75% of family members diagnosed with MS harboring these substitutions, and thus not fulfilling our criteria for segregation with disease (S2 Table). Although p.Pro462Thr did not co-segregate with MS, it was genotyped in MS probands and controls from Canada, as it is located three amino acids C-terminal from p.Gly459Asp, has not been previously described, is predicted damaging for protein function (CADD = 27.1), and is evolutionarily conserved (S1A Fig). This additional genotyping effort did not identify any additional carriers, thus assessment of p.Pro462Thr in additional MS cohorts and multi-incident families is necessary to define its role in MS.

MASP1 is a serine protease of the lectin pathway that triggers complement activation by cleaving C2, and forming the C3 convertase C4b2a (Fig 3). *MASP1* encodes three different transcripts; isoform 1 is the longest and is composed of a heavy chain and a light chain containing the serine protease domain, isoform 2 has the same heavy chain as isoform 1 but a different light chain serine protease domain, and isoform 3 is composed of a single shortened heavy chain [37]. The p.Gly459Asp substitution identified in MS patients is located exclusively in the serine protease domain of isoform 2, also known as MASP3, is highly conserved in orthologs and most paralogs (Fig 4), and is predicted damaging for protein function (CADD = 26.2). MASP3 is thought to regulate complement activation by inhibiting MASP2 function in the lectin pathway, and promoting factor D (FD) activation in the alternative pathway [38, 39]. Recessive loss of function mutations in MASP1, resulting in impairment of MASP3, cause 3MC syndrome; a rare disorder characterized by facial dysmorphism, and commonly presenting cleft lip and palate, postnatal growth deficiency, hearing loss, and cognitive impairment [37, 40]. In MS patients with active disease, serum levels of MASP3 have been shown to inversely correlate with MASP2 levels and the pathological antibody response to herpes virus, leading the authors to suggest a protective role for MASP3 in MS [41].

#### Complement component 2 (C2)

We identified a large family with five relatives diagnosed with MS (Fig 2). To identify the genetic factor responsible for the onset of disease, we performed WES analysis in four family members presenting MS symptoms (IV-1, 2, 6, 7). This analysis failed to identify a rare heterozygote missense or nonsense variant shared amongst all affected individuals; however, segregation analysis for variants observed in any three of the four family members who underwent WES analysis identified a rare variant in C2 (p.Thr184Met) co-segregating with disease. The variant was identified in four family members diagnosed with MS (III-2, IV-1, 6, 7), one out of six unaffected family members (III-3), and one obligate carrier (III-6). Genotyping C2 p.Thr184Met in MS patients and controls did not identify any additional carriers (Table 1). The mean age at onset of MS for family members carrying the C2 p.Thr184Met substitution is 33.3 years (SD ± 4.0). The clinical disease course for C2 p.Thr184Met carriers was RRMS, which only became progressive for IV-7 (S1 Table). The level of disability is quite variable with some family members (IV-6) presenting a EDSS of 2.5 after 7 years of disease, while others (III-2) were deceased at 47 years of age, 16 years after the onset of MS. Analysis of exome data from 441 MS patients identified a rare C2 p.Lys526Arg variant not present in 1,055 WES controls; however, genotyping C2 p.Lys526Arg in nine additional family members diagnosed with MS did not support co-segregation with disease, and was excluded from further analysis (S2 Table).

In the complement activation cascade, C2 binds to surface-bound C4b and is cleaved into C2a and C2b by activated factor C1s in the classical pathways or MASP1/MASP2 in the lectin pathway (Fig 3). C2b is then released whereas C2a remains attached to C4b providing the catalytic subunit for C3 or C5 convertases [39]. The p.Thr184Met variant, which is conserved in mammals (Fig 4), and predicted damaging for protein function (CADD = 22.2), is located in the third complement control protein (CCP) module of C2, and disrupts its third β-strand segment. CCP domains are evolutionary conserved structures commonly found in proteins of the complement system but also present in many non-complement proteins. CCP domains are typically composed of five β-strand segments that run back or forth forming a β-sandwich surrounding a hydrophobic core [42]. Although all CCP modules share a common tertiary structure, their biological function is diverse. The CCP domains in C2 are involved in C3b and C4b binding [43], thus the p.Thr184Met substitution identified in this family could potentially affect the kinetics of the complement cascade activation, by disrupting the formation of C3 and C5 convertases, altering the production of anaphylatoxins C3a and C5a, and the formation of the membrane attack complex.

Complement components have been linked to MS, with cerebrospinal fluid (CSF) of patients presenting increased C3 levels compared to controls, particularly those diagnosed with primary progressive (PP)MS. C3 levels were also found to correlate with degree of clinical disability [44], and an association between a functional variant in C3 (p.Arg102Gly) and cognitive impairment, brain atrophy and greater lesion burden in MS patients has been described [45]. Similarly, anaphylatoxin C3a and C5a receptors are upregulated in patients with MS [46], and increased complement anaphylatoxin receptor-positive microglia in progressive MS patients has been suggested as a source of sustained neuroinflammatory response driving myelin and neuronal pathology [27]. In addition, mouse models of MS have shown C3a and C5a to contribute to demyelination, delayed remyelination, worsen disease severity, and being capable of inducing the production of pro-inflammatory cytokines and chemokines [47, 48].

### Inflammasome assembly

An inflammasome is a cytosolic protein complex that is critical for secretion of interleukin (IL)-1β and IL-18, initiating an inflammatory cascade and inducing pyroptosis. Although the majority of studies support a central role for inflammasomes in the innate immune response, a role in T-cell biology has also been suggested. The assembly of the inflammasome is activated by pattern-recognition receptors (PRR) sensing pathogen-associated molecular patterns (PAMPs) and danger-associated molecular patterns (DAMPs), or changes in intracellular cation concentrations [49]. Several PRR sensor molecules can activate inflammasome complex formation, and include nucleotide-binding domain (NOD or NACHT)-leucine rich repeat (LRR)-pyrin domain (PYD)-containing proteins (NLRPs) and NATCH-LRR-caspase activation and recruitment domain (CARD)-containing proteins (NLRCs) [50]. Each NOD-like receptor (NLR) is activated by unique stimuli and promote the formation of a specific inflammasome. The assembly of the inflammasome complex serve as a scaffold for the recruitment of the apoptosis-associated speck-like protein containing a CARD (ASC) adaptor, encoded by *PYCARD*, and oligomerization of the inactive zymogen pro-caspase-1, initiating its autoproteolytic cleavage and activation (Fig 3). Caspase-1 then cleaves cytokine precursor pro-IL-1β and pro-IL-18 into their biologically active forms which are secreted and trigger a potent inflammatory response [51, 52]. A subgroup of NLRs, including NLRP12, NLRC3 and NOD2 are capable of enhancing or attenuating inflammatory signaling cascades by modulating diverse signaling pathways, including the NF-κB and extracellular signal-regulated kinase (ERK) pathways, which regulate the expression of inflammasome components, cytokines and chemokines [49, 53].

Mutations in several inflammasome components can cause autoinflammatory syndromes. Activating mutations in *NLRP3* cause cryopyrin-associated periodic syndromes (CAPS), which is characterized by systemic inflammation with fever and blood neutrophilia [52]. Interestingly some low penetrance *NLRP3* CAPS mutations have been described in patients diagnosed with MS, suggesting a role for the inflammasome in the onset of autoimmune diseases [54]. This is further supported by studies showing an increased expression of NLRP3 inflammasome-related genes in RRMS patients compared to controls [55]. In addition, the study of multi-incident MS families have nominated pathogenic mutations in *NLRP1*, and purinergic receptors *P2RX4*/*P2RX7* which initiate inflammasome formation by modifying intracellular calcium and potassium concentrations [10, 11]. Rare missense variants in *NLRP5* and *NLRP9* have also been found to correlate with disease course and severity in MS patients [8, 56].

#### NLR family pyrin domain containing 12 (NLRP12)

To identify the genetic cause for the onset of MS in a large multi-incident family with six siblings diagnosed with MS, we performed WES in four affected family members (II-1, 2, 4, 5). This analysis identified a NLRP12 p.Leu972His substitution segregating with disease in all six family members diagnosed with MS (Fig 2). Detailed clinical information is available for four patients, and suggests that the clinical course for NLRP12 p.Leu972His is progressive MS, with II-1 and II-2 presenting PPMS, II-4 RRMS with incomplete remission and II-5 SPMS (S1 Table). The mean age at onset of MS was 29.4 years (SD ± 5.1).

The p.Leu972His substitution, which has not been previously described, and was not identified in any additional samples, affects one of the seven amino acids that are critical for the formation of the sixth LRR domain of NLRP12 (Fig 5B) [57, 58]. The mutated leucine is conserved in orthologs and most human paralogs (Fig 4) highlighting the importance of this residue for protein function (CADD = 24.9). LRR domains in PRRs are thought necessary for the recognition of PAMP and DAMP ligands, and to maintain an auto inhibited state, as the deletion of LRR domains generally leads to constitutively active proteins [59].

Mining exome data from 441 MS patients identified a rare p.Arg352Cys in two blood-related patients from Italy, albeit not segregating with disease in additional family members (S2 Table), and a p.Leu475Gln substitution in an affected sibling pair from Canada (Fig 2). In this family, the age at onset of MS was 39 and 22 years for II-1 and II-2, respectively. II-1 presented a clinical course consistent with PPMS and a EDSS of 7.5 after 4 years of disease. In contrast the clinical course for II-2 was consistent for RRMS without apparent gait impairment (EDSS = 0) (S1 Table). Screening MS patients and controls for NLRP12 p.Leu475Gln did not identify any additional carriers (Table 1). The p.Leu475Gln substitution, which is conserved in orthologs (Fig 4) and has not been previously described, is located in the NACHT domain of NLRP12. NACHT domains have ATPase activity and are thought to be important for protein oligomerization [49]. Interestingly, the recessive NLRP1 p.Gly578Ser mutation described in a sibling pair diagnosed with MS, and shown to increase IL-1β expression, is also located in the NACHT domain and only seven amino acids N-terminal from the homologous p.Leu475 in NLRP12 (Fig 4) [11].

Mutations in *NLRP12* have been described in patients with familial cold autoinflammatory syndrome (FCAS), that causes symptoms similar to CAPS with fever, myalgia and elevated serum inflammatory markers [52]. Interestingly the p.Arg352Cys substitution identified in one Italian family has been associated with FCAS (S2 Table) [60, 61]. To assess whether this variant also associates with MS, we genotyped 2,502 MS patients and 1,075 healthy controls from Canada. This analysis identified NLRP12 p.Arg352Cys in one control and one familial proband, however segregation did not support pathogenicity (Tables 1 & S2). The allelic frequency observed in unrelated MS patients (0.02%) and healthy controls (0.05%), together with lack of co-segregation in MS families, does not support a role for p.Arg352Cys in MS.

NLRP12 knock-out mice were found to present hyperactivated T-cells and increased expression of prostaglandin-endoperoxide synthase 2 (COX2), C-C motif chemokine receptor 5 (CCR5) and IL-1β. In addition, lipopolysaccharide (LPS) stimulation in primary microglial cells from NLRP12 knock-out mice triggered a significant increase in the expression of nitric oxide synthase (iNOS), tumor necrosis factor (TNF-α) and IL-6, compared to wild-type cells [62]. The induction of EAE in these mice has however provided conflicting outcomes, with some studies reporting exacerbated clinical phenotype while others describe reduced disability with atypical symptoms [62, 63].

Transfection of microglial BV2 cells with wild-type, p.Gln475 or p.His972 NLRP12 showed significant differences in caspase-1 (ANOVA, *p* = 4.3×10^−6^) (Fig 5C). Wild-type and p.Gln475 NLRP12 caused an almost two-fold increase in caspase-1 activation compared to empty vector (Tukey’s, *p* = 5.8×10^−5^, *p* = 2.2×10^−5^), whereas no differences were observed for p.His972 (Tukey’s, *p* = 1.0). Using a luciferase reporter assay, we also ascertained the inhibitory effect of NLRP12 on NF-κB activation, which showed no differences between wild-type and mutant proteins (S2 Fig). Taken together, these data suggest impaired inflammasome activation as the mechanism of disease for NLRP12 p.Leu972His. In contrast, lack of differences between p.Leu475Gln and wild-type NLRP12 suggest that this variant is a rare non-pathogenic polymorphism, or has an alternative yet to be defined mode of action.

### Wnt signaling

The Wnt signal transduction pathway regulates multiple biological processes including cell proliferation, migration, polarity, differentiation and axon outgrowth. Wnt proteins have also been shown to regulate effector T-cell development, regulatory T-cell activation and dendritic-cell maturation, and to play an important role in the expression of inflammatory mediators during bacterial infections [64, 65]. At least three Wnt-dependent pathways have been proposed; one canonical Wnt/β-catenin pathway, and two non-canonical pathways, which include the Wnt/Ca^2+^ pathway activated through the nuclear factor of activated T-cells (NFAT). Activation of the canonical pathway is initiated through binding of Wnt ligands to Frizzled (FZD) receptors, causing an accumulation of β-catenin in the cytosol and subsequent translocation to the nucleus, where it forms an active transcription factor complex with T-cell factor/lymphoid enhancer factor (TCF/LEF) [64, 66]. Activation of the non-canonical Wnt/Ca^2+^ pathway by FZD receptors triggers calcium release from intracellular stores. Increased intracellular calcium concentrations in turn activates, calcineurin, which dephosphorylates NFAT unmasking the nuclear localization sequence, facilitating nuclear translocation and activation of Wnt/Ca^2+^ target genes (Fig 3) [67].

Wnt signaling in microglial cells induces a strong pro-inflammatory response through the activation of the canonical Wnt/β-catenin pathway. This activation mediates increased expression of cytokines, including IL-6, IL-1α and IL-15, chemokines such as C-X-C motif chemokine ligand 2 (CXCL2), CXCL11, and C-C motif chemokine ligand 7 (CCL7), innate immune response components including complement C3, and inflammasome components NLRP3 and NOD2 [68]. The Wnt/β-catenin pathway is also a major key signaling mechanism for myelinating processes, as well as oligodendrocyte development and differentiation [69–71]. The canonical Wnt pathway is also required for angiogenesis in the CNS, maturation of the blood-brain-barrier (BBB), and reduced immune cell infiltration [72, 73]. Activation of the Wnt/Ca^2+^ pathway regulates cytokine production in T-cells, but it is also integral for T-cell proliferation, differentiation, and activation [74]. Activation of both, canonical and non-canonical Wnt pathways, is protective against neurotoxic injury and has been found to be deregulated in degenerative and inflammatory CNS disorders [75]. In the EAE model of MS, activation of the canonical Wnt signaling pathway promotes neurogenesis and repair, whereas its inhibition results in exacerbated clinical scores [73, 76]. In contrast, activation of the Wnt/Ca^2+^ pathway in EAE mice triggers an amplified pro-inflammatory response [77].

#### Ubiquitin protein ligase E3 component N-recognin 2 (UBR2)

The analysis of a multi-incident family with four family members diagnosed with MS led to the identification of a rare missense variant in UBR2 (p.Ala1658Thr) segregating with disease (Fig 2). This variant, which has not been previously described, was identified in all four family members diagnosed with MS, one obligate carrier (II-5) and one unaffected family member (III-3). The clinical disease course for this family appears to be mostly progressive with three family members (II-3, III-2, 4) presenting PPMS at the onset of disease, and one with RRMS (II-7) (S1 Table). The mean age at MS onset was 37.5 years (SD ± 7.4) with a range of 29 to 47 years. The identification of two carriers without clinical symptoms of MS at 52 (III-3) and 55 (II-5) years of age, suggests that UBR2 p.Ala1658Thr has reduced penetrance. No clinical information is available for II-2, who died at 47 years of age.

Although the substitution identified in UBR2 is not located in a known functional protein domain, it is evolutionarily conserved in orthologs and one of two paralogs (Fig 4). UBR2 p.Ala1658Thr is also predicted deleterious on protein function with a CADD score of 32.0. Genotyping of cases, controls and Italian families for UBR2 p.Ala1658Thr failed to identify any additional carriers (Table 1). Mining WES data identified three rare UBR2 variants in MS probands that were absent in WES controls; however, genotyping of additional family members showed insufficient co-segregation with MS to support pathogenicity (S2 Table).

UBR2 encodes an E3 ubiquitin ligase of the N-end rule proteolytic pathway, and targets proteins with destabilizing N-terminal residues for ubiquitination and proteasome-mediated degradation. Although its mechanism of action still remains to be elucidated, UBR2 has been shown to regulate the activation of the Wnt/β-catenin pathway [78].

#### Catenin alpha 3 (CTNNA3)

We identified a family with five members diagnosed with MS (Fig 2), and analysis of WES data from III-3, III-11, and III-12 uncovered a CTNNA3 p.Ala852Ser substitution co-segregating with MS in all affected individuals. This variant was also identified in four obligate carriers and one of seven unaffected family members (III-5), who was 48 years of age at examination. The age at onset of MS in this family presents a broad range (15–39 years) with a mean of 26.6 years (SD ± 8.8), with two patients presenting PPMS (III-10, 12) and three RRMS (III-3, 6, 11) clinical course (S1 Table). Disease severity appears to be relatively mild for III-10 and III-11 with EDSS of 3.5 and 1.5 after 25 and 9 years of disease, respectively. In contrast III-12 presented a EDSS of 7.0, 24 years after the onset of MS. Genotyping of CTNNA3 p.Ala852Ser in additional samples did not identify any more carriers (Table 1). Mining WES data from MS patients identified six rare missense substitutions not observed in WES controls (S2 Table). However, assessment of segregation within families did not support a role for these variants in the onset of MS.

The p.Ala852Ser substitution occurs not only in a residue conserved in protein orthologs and α-catenin paralogs CTNNA1 and CTNNA2, but also in a highly evolutionarily conserved protein region (Fig 4). Despite this high level of conservation and the predicted damaging effect on protein function (CADD = 26.2), this substitution is not known to affect a defined protein domain or motif.

CTNNA3 encodes α-T-catenin; one of the critical mediators of the cadherin/catenin adhesion complex, the major cell-cell adhesion system in the body [79]. Although α-T-catenin is predominantly expressed in the heart and testis, lower expression has been observed in other tissues including brain; specifically in the cytoplasm of neurons, neurite projections, and adherens junctions bordering active synapses [79–81]. Interestingly, α-catenins also participate in Wnt signaling, with overexpression of α-T-catenin causing impaired activation of the β-catenin pathway [81].

Mutations in *CTNNA3* have been identified in families with arrhythmogenic right ventricular dysplasia, autism spectrum disorder, and several carcinomas [79, 82, 83]. In addition, genetic associations have been described with steroid resistant asthma and inflammation, essential tremor, and controversially Alzheimer’s disease [84–86]. A suggestive genetic association between *CTNNA3* and MS has been described (*p* = 0.001), but did not withstand multiple testing correction [6]. Given the broad range of phenotypes ascribed to *CTNNA3*, it is important to note that obligate carrier II-7 developed bronchitis, cancer and osteoporosis, whereas III-10 and III-11 developed cancer and rheumatic fever, respectively, in addition to MS.

#### Nuclear factor of activated T-cells 2 (NFATC2)

WES was performed in three family members diagnosed with MS (III-4, 10, IV-3) from a large multi-incident family with seven affected individuals. This analysis led to the identification of a novel NFATC2 p.Pro679Leu substitution co-segregating with MS (Fig 2). It should be noted that one family member (III-9) has conflicting clinical reports, and the affection status is unverified. Detailed clinical information was available for four family members diagnosed with MS (S1 Table). III-2 and III-4 presented a clinical course consistent with PPMS, the latter with a disability score of 6.5 after 44 years of disease. In contrast, IV-3 and IV-4 were diagnosed with RRMS and with a EDSS of 1.5 after five and one year of disease duration, respectively. The mean age at onset of MS for NFATC2 p.Pro679Leu carriers was 33.0 years (SD ± 8.3). Genotyping 2,502 MS patients, 1,075 controls, and 15 Italian families for this highly conserved (Fig 4) and predicted damaging (CADD = 27.1) substitution, did not identify any additional carriers. Mining WES data from MS patients revealed five rare missense variants, however segregation analysis within families did not support pathogenicity (S2 Table).

NFATC2 is one of several transcription factors regulated by calcineurin in the Wnt/Ca^2+^ signaling pathway that mediates T-cell function, oligodendrocyte differentiation, and coordinates the induction of cytokines and immunoregulatory molecules [87–89]. Calcineurin activation is thought to be dependent on calcium release from intracellular compartments; however, increased expression of NFAT target genes has been observed following extracellular calcium influx through transport and cation exchange channels [90, 91].

The study of infection and allergy in NFATC2 knockout mouse models showed enhanced immune responses, with increased cytokine production in peripheral T-cells [92]. In contrast, NFATC2 deficiency in mast cells resulted in a strong reduction in cytokine production, and delayed inflammatory response; indicating cell-specific functions [93, 94]. The induction of EAE in NFATC2 knockout mice causes a markedly reduced clinical score, compared to wild-type animals. This protective effect was shown to be mediated by a differential cytokine expression in CD4^+^ T-cells, and led to the suggestion of NFAT repression as a potential DMT for MS [95]. These studies would also suggest that the mechanism of action for the p.Pro679Leu substitution is mediated through increased NFATC2 activity.

#### Ring finger protein 213 (RNF213)

The analysis of five sisters diagnosed with MS led to the identification of a rare RNF213 p.Asn2327Asp substitution segregating with disease (Fig 2). The mean age at onset of MS in this family is 31.2 years (SD ± 5.4). Detailed clinical information was available for four patients (II-1, 3, 5, 6), all presenting RRMS course at the onset of disease (S1 Table). Three family members (II-1, 3, 6) have mild symptoms, with EDSS between 0 and 1.5 and without signs of progressive MS after 3, 8 or 12 years of disease. In contrast, II-5 developed SPMS five year after the onset of MS, had a EDSS of 9 after 15 years of disease duration, and was deceased five years later at 44 years of age. The variant identified in these five sisters was inherited from their mother (I-2), who at 68 years of age disclosed not to be suffering from MS symptoms.

Genotyping of RNF213 p.Asn2327Asp in 2,502 MS patients and 1,075 healthy controls from Canada identified this variant in eight additional familial probands and four patients without a family history of MS, resulting in a MAF of 0.26%. Segregation analysis in these additional families identified the variant in 16 out of 21 MS patients and 10 of 21 unaffected family members (excluding parents of MS patients). Although the proportion of MS patients harboring the RNF213 p.Asn2327Asp substitution is over the 75% threshold selected for positive co-segregation with disease, two patients without a family history of MS were found to have three or four unaffected siblings carrying the variant; thus not fulfilling our criteria for pathogenicity (Fig 2). Although no unrelated healthy controls were found to harbor RNF213 p.Asn2327Asp in our cohort, this substitution has been observed in populations of European descent with a MAF of 0.13% [17]. Although the data from this publicly available database has been generated primarily from diseased individuals, not healthy controls, and from a locale geographically distinct to our MS cohort [96]; it suggests that RNF213 p.Asn2327Asp is a risk factor for MS, with carriers having more than twice the risk of developing disease (OR = 2.07; 95% CI = 1.15–3.72).

Mining WES data from 441 MS patients identified one nonsense and 16 missense variants in RNF213 with a reported MAF below 1%, and absent in WES data from control samples (Tables 1 & S1). Genotyping additional family members provided evidence against pathogenicity for all variants except RNF213 p.Ile2446Thr, which was only observed in one patient and one unaffected sibling (Fig 2). Although one additional sibling diagnosed with MS was known to exist in this family, a DNA sample was not available for study. Genotyping Canadian MS patients and controls for RNF213 p.Ile2446Thr did not identify any additional carriers, thus not providing any additional evidence for or against a role in MS. However, given that this variant is not evolutionarily conserved, it may represent a rare benign polymorphism (Fig 4).

RNF213 is an ATPase and E3 ubiquitin ligase protein which targets NFATC2 for proteasomal degradation, attenuating the non-canonical Wnt/Ca^2+^ pathway (Fig 3). As a result, RNF213-deficiency has been shown to trigger an increased expression of NFAT target genes [97]. Elevated activity of the Wnt/Ca^2+^ pathway has also been observed in patients with Moyamoya disease (MMD), which is caused by missense mutations in *RNF213* [98]. MMD, a cerebrovascular disease characterized by progressive occlusive or stenotic arterial lesion, is one of the major causes of stroke in adults and children worldwide. The variant most commonly associated with MMD in East Asian populations is RNF213 p.Arg4810Lys, which has been shown to modulate cerebral blood flow through angiogenesis [99]. This mutation has incomplete penetrance resulting in a spectrum of clinical phenotypes, termed quasi-MMD, and typically include intracranial atherosclerosis and autoimmune diseases such as Grave’s disease, autoimmune thyroid disease, rheumatoid arthritis, psoriasis, and autoimmune gastritis [100]. A variant associated with MMD in European populations (p.Arg4019Cys) was identified in two Canadian families analyzed in this study [101]. Given its association with MMD and the described link between *RNF213* and autoimmune diseases, we genotyped p.Arg4019Cys in MS patients and controls from Canada (Table 1). This analysis identified three additional families harboring the RNF213 p.Arg4019Cys substitution (S1B Fig). Although assessment of segregation does not support pathogenicity, the identification of this substitution in five MS families and no controls warrants further studies to elucidate whether RNF213 p.Arg4019Cys causes a low-penetrance phenotype with a clinical presentation of MS.

### Nuclear receptor complexes

Nuclear receptors are ligand-activated transcription factors that play integral roles in many physiological processes by directly regulating gene expression. These processes include metabolism, immunity, homeostasis, cell proliferation and development, amongst others [102]. In general, nuclear receptors bind to promoter-specific DNA sequences and interact with co-repressor complexes to inhibit gene expression. Ligand-induced activation of nuclear receptors triggers the dissociation of inhibitory complexes, and the recruitment of nuclear receptor co-activator complex components that promote gene transcription [102].

Amongst others, the nuclear receptor family of transcription factors include vitamin D receptor (VDR), peroxisome proliferator activated receptors (PPARs), and liver X receptors (LXRs), which have been shown to play important roles in the pathophysiology of MS. VDR is expressed in immune cells, and modulates the innate and adaptive immune responses. In addition, Vitamin D insufficiency is common in MS patients, and was found to correlate with disease activity, disability, and progression [103, 104]. The activation of PPAR and LXR have been shown to inhibit canonical and non-canonical Wnt pathways, and the NF-κB signaling pathway; resulting in dysregulated inflammatory response and impaired remyelination [69, 105–107]. These findings are supported by studies in the EAE model of MS, which showed that PPAR and LXR-deficient mice presented an exacerbated clinical phenotype, higher cytokine production, and more severe demyelination compared to wild-type or untreated animals [107–110]. In addition, LXR-α which is encoded by *NR1H3*, was found to harbor a rare p.Arg415Gln mutation co-segregating with MS in two multi-incident families, and common alleles resulting in increased disease susceptibility [9]. Although this association was initially controversial [96, 111], it has now been independently replicated [6, 112].

#### Nuclear receptor coactivator 3 (NCOA3)

Exome sequencing in four family members diagnosed with MS nominated NCOA3 p.Arg485Cys as the genetic factor responsible for the onset of MS in this multi-incident family (Fig 2). Genotyping NCOA3 p.Arg485Cys in additional samples identified two patients from Canada harboring this variant. Segregation analysis in these families, with only one exception (III-13), identified NCOA3 p.Arg485Cys in all patients diagnosed with MS. In addition, three unaffected family members and four obligate carriers, were also found to present this variant (Fig 2). Interestingly, these three families are not ancestrally related, suggesting that this position is a mutational hotspot (S3A Table). Clinical information for variant carriers is limited, but suggest that NCOA3 p.Arg485Cys patients developed MS on average at 27.8 years of age (SD ± 5.6), predominantly with a progressive disease course, similar to patients with *NR1H3* mutations [9]. Disability appears to be severe for II-8 and mild for III-10, who is the only family member known to present RRMS (S1 Table). With the exception of IV-5, who was 24 years old at examination, all other healthy NCOA3 p.Arg485Cys carriers are markedly older than the mean age at onset for MS carriers, with ages between 41 and 82 years at examination. Mining WES data from MS patients identified seven additional rare variants causing NCOA3 substitutions; however, segregation analysis does not support a role for any of these variants in MS (S2 Table).

Given the number of unaffected carriers in NCOA3 p.Arg485Cys families, we genotyped this variant in a European cohort consisting of 3,752 MS patients and 2,803 healthy controls from Spain and Austria to further define its role in MS. This analysis identified three additional carriers of NCOA3 p.Arg485Cys; two male patients diagnosed with RRMS at 24 and 34 years of age, and one healthy female. The allele frequency in the Canadian, Spanish and Austrian populations combined is 0.04% for MS patients (5/6,252) and 0.01% for healthy controls (1/3,877), suggesting that individuals with the NCOA3 p.Arg485Cys substitution have a 3.1-fold increased risk of developing MS (95% CI = 0.36–26.56). This data is further supported by a strongly suggestive association observed between common genetic variants in *NCOA3* and MS risk (*p* = 5.78×10^-5^, OR = 1.1) [6].

Although p.Arg485Cys does not occur within a known NCOA3 domain, the affected arginine residue is evolutionarily conserved in orthologs and paralogs (Fig 4), and the substitution predicted damaging for protein function (CADD = 26.8). Preliminary characterization of NCOA3 p.Cys485 showed a 3.5-fold increased expression of iNOS in microglial cells transfected with mutant protein (ANOVA, *p* = 3.7×10^−5^) compared to cells transfected with wild-type NCOA3 (Tukey’s, *p* = 7.5×10^−5^) or empty vector (Tukey’s, *p* = 1.4×10^−4^) (Fig 5D). Thus suggesting that NCOA3 p.Arg485Cys results in gain of function that triggers a pro-inflammatory response under basal conditions.

NCOA3, also known as steroid receptor co-activator 3 (SRC3), is a transcriptional co-activator of nuclear receptor complexes, including PPAR and LXR, that recruits histone acetyltransferases and methyltransferases for chromatin remodeling and activation of gene expression [113, 114]. NCOA3 has been shown to be involved in inflammatory responses, and to play an important role in innate immunity and maintenance of T-cell function [115]. The induction of EAE in NCOA3-deficient mice causes attenuated disease severity, decreased inflammation and CNS infiltration, and reduced demyelination compared to wild-type animals. This neuroprotective effect was shown to be mediated by PPAR-β upregulation, which induced microglial expression of anti-inflammatory cytokines, opsonins, and neurotrophic factors [114].

The identification of ten patients diagnosed with MS harboring a rare NCOA3 p.Arg485Cys substitution capable of enhancing the expression of pro-inflammatory mediators, together with a suggestive association between MS and *NCOA3*, and significant associations with *NCOA1*, *NCOA5*, *NR1D1* and *NR1H3* [6, 9], suggests an important role for nuclear receptor co-activators, and the LXR and PPAR pathways, in the pathophysiology of MS.

### Cation channels and exchangers

Compared to the extracellular medium, most mammalian cells have low concentrations of sodium and calcium, and large concentrations of potassium ions. This cation imbalance is regulated by membrane permeability and ion exchangers, which are critical to maintain cellular homeostasis. In cells of the innate and adaptive immune systems, ion channels and ion transporters modulate membrane potentials and regulate several physiological functions, including gene expression, apoptosis, proliferation, and migration [116]. Oscillations in intracellular calcium concentrations, due to an intricate interplay between calcium, potassium, sodium and chloride channels in the plasma membrane as well as intracellular organelles, regulate the function of many enzymes and transcription factors implicated in lymphocyte development, innate and adaptive immune responses, and autoimmunity [116]. A role for ion transporters in the pathophysiology of MS is supported by upregulation of calcium and potassium channels in MS patients, triggering apoptotic signals, demyelination and neuronal degeneration [117]. In addition, significant associations with MS risk, and pathogenic mutations have been described in *P2RX4* and *P2RX7*, non-selective cation channels activated by extracellular ATP [10, 118], and a missense variant in calcium voltage-gated channel subunit alpha1 H (*CACNA1H*) was found nominally associated with MS clinical course [8]. Small molecules capable of modulating voltage-gated calcium, sodium, and potassium channels have been developed to treat pain, stroke, migraine, epilepsy, cancer, and autoimmune disorders amongst others; and are thought to provide a good basis for the development of novel MS treatments [119–121].

#### Potassium voltage-gated channel modifier subfamily G member 4 (KCNG4; Kv6.4)

Exome sequencing analysis in three MS patients (III-2, 4, 9) from a large multi-incident family led to the identification of a KCNG4 p.Arg474His substitution co-segregating with disease (Fig 2). This variant was identified in six out of seven family members diagnosed with MS, and no unaffected family members. The mean age at onset of MS for KCNG4 p.Arg474His carriers is 21.6 years (SD ± 5.0), and typically present a RRMS course with mild disability (S1 Table). The clinical presentation for III-9 is quite distinct from the other family members, as she developed PPMS at 14 years of age, had a EDSS of 9 after 34 years of disease duration, and was deceased by 54 years of age. Genotyping of KCNG4 p.Arg474His in MS cases and controls from Canada and Italian families did not identify any additional carriers (Table 1). Mining of exome data identified three rare arginine to histidine substitutions (p.Arg208His, p.Arg343His, p.Arg365His) in MS patients not present in WES control samples. Segregation analysis for p.Arg208His and p.Arg343His did not fulfil our criteria for co-segregation with disease (S2 Table). In contrast, KCNG4 p.Arg365His was identified in two family members diagnosed with MS and only one of five unaffected family members (Fig 2), thus co-segregating with MS. No additional p.Arg365His carriers were identified in MS cases or controls. In contrast to p.Arg474His, patients harboring the p.Arg365His substitution developed MS at an older age (49 and 37 years for III-3 and III-5, respectively), with PPMS disease course, and a higher level of disability (EDSS of 6 and 7, after 8 and 21 years of disease duration) (S1 Table). III-1, who also carries the KCNG4 p.Arg365His substitution, was interviewed at 65 years of age and did not report suffering MS.

The arginine residues replaced by p.Arg365His and p.Arg474His are evolutionarily conserved in most orthologs and paralogs (Fig 4), and the substitutions predicted damaging to protein function (Table 1). Voltage-gated potassium (Kv) channels are selective membrane proteins, forming a tetrameric complex activated in response to changes in membrane potential. *KCNG4* encodes Kv6.4, a silent Kv subunit that requires assembly with Kv2 subunits to form functional heterotetramers. Each Kv subunit contains six transmembrane segments; 1 to 4 form the voltage-sensing domain and segments 5 and 6 form the central pore structure. Activation of Kv channels is thought to be regulated by dynamic coupling of the cytoplasmic linker between transmembrane segments 4 and 5 and the lower half of segment 6 [122]. Interestingly, KCNG4 p.Arg365His is located in the regulatory linker between transmembrane segments 4 and 5, and p.Arg474His in the cytoplasmic C-terminal domain, 13 amino acids from transmembrane segment 6.

The biological properties for KCNG4 are yet to be resolved; however, a role for potassium channels in MS is supported by increased expression of several Kv channels in inflammatory infiltrates, and demyelinated lesion areas and axonal segments in pathological samples from MS patients [123, 124]. In addition, blocking the activity of Kv channels has been shown to suppress the immune response, and reduce severity in EAE mice by preventing demyelination and axonal degeneration [124, 125]. Potassium channels have also been postulated as potential targets for the development of immunomodulatory therapies for MS, and some non-selective potassium channel inhibitors have been approved for the treatment of MS patients [126, 127].

#### Solute carrier family 24 member 1 (SLC24A1; NCKX1)

The analysis of WES data from three family members diagnosed with MS (II-2, III-1, 2) nominated a SLC24A1 p.Leu26Phe substitution as the putative genetic factor responsible for the onset of disease. This variant was identified in all six family members diagnosed with MS and none of their unaffected siblings (Fig 2). Genotyping SLC24A1 p.Leu26Phe in Italian families and MS patients and controls from Canada identified two additional carriers diagnosed with MS (Table 1), and assessment of segregation in blood relatives from these probands (III-4 and II-14) identified the variant in one additional family member diagnosed with MS (II-10) and one unaffected sibling (III-5) who was 63 years of age at last examination (Fig 2). One family member (III-9) diagnosed with MS not harboring this variant, was also identified. In total, nine patients from three families were found harboring the SLC24A1 p.Leu26Phe substitution. Haplotype analysis shows that these families are not ancestrally related, suggesting that at least three independent mutational events resulting in the same p.Leu26Phe substitution have occurred (S3B Table). The mean age at MS onset for SLC24A1 p.Leu26Phe carriers is 31.8 years (SD ± 10.0), with a clinical course consistent with RRMS (II-9, III-2, 3, 4), PPMS (II-2, 4, III-1) or SPMS (II-14) (S1 Table). Mining WES data from MS patients identified one rare nonsense and four missense variants not present in controls; however, segregation analysis within additional family members does not support a role for these variants in MS (S2 Table).

*SLC24A1* encodes a sodium/calcium, potassium exchanger (NCKX1) that removes intracellular calcium by exchanging four sodium ions for one calcium and one potassium [128]. The p.Leu26Phe substitution identified in MS patients is part of an uncleaved signal peptide sequence required for efficient membrane targeting and integration [129]. This signal sequence is highly conserved in mammals, including the p.Leu26 residue (Fig 4), but no homologous region exist in paralogs. The substitution of leucine for phenylalanine is predicted moderately damaging for protein function (CADD = 15.9), but further analyses are necessary to address whether p.Phe26 disrupts SLC24A1 membrane integration and protein conformation.

Although *SLC24A1* is thought to be most highly expressed in retinal rods photoreceptors, it is also expressed in other cell types, including cells of the immune system [130]. In retinal rods, SLC24A1 is the principal extruder of calcium ions during light adaptation, and recessive mutations have been described in families presenting congenital stationary night blindness (CSNB1D), a non-progressive retinal disorder associated with impaired night vision [131, 132]. Limited information is currently available for SLC24A1 in other cell types; however in the EAE model, several calcium channel blockers have been shown to reduce cytokine production, inflammation and axonal pathology, while promoting remyelination [133–135].

#### Solute carrier family 8 member B1 (SLC8B1; NCLX)

The analysis of WES data from three cousins (III-2, 3, 7) diagnosed with MS, identified a rare missense variant in SLC8B1 (p.Ser94Gly) segregating with disease (Fig 2). This substitution was identified in four out of five family members diagnosed with MS, and none of the unaffected siblings. A single rare protein-altering variant shared amongst all five family members diagnosed with MS could not be identified. One additional family member diagnosed with MS was known to exist (III-8), but DNA was not available for study. Genotyping of SLC8B1 p.Ser94Gly in MS patients and healthy controls from Canada and Italian families did not identify any additional carriers (Table 1). On average, the age at onset of MS for patients harboring the SLC8B1 p.Ser94Gly substitution was 29.8 years (SD ± 13.5), although one family member is a clear outlier (III-5). This patient was suspected of suffering from MS for many years, but confirmation was only obtained at 50 years of age, more than a decade after the initial presentation of clinical symptoms (S1 Table). Detailed clinical course is available for III-2 and III-7, and they both presented RRMS at the onset of disease. Thirty-two years after the onset of MS, the clinical course for III-2 was SPMS with a EDSS of 6.5; in contrast, III-7 continued to present RRMS after 22 years of disease, with a EDSS of 6.0.

Analysis of *SLC8B1* in WES data from Canada and Italy identified one nonsense and four missense variants in MS patients not observed in WES controls, and with a MAF below 1% in publicly available databases. Segregation analysis in additional family members did not support a role for any of the missense substitutions in the onset of MS (S2 Table). In contrast, the nonsense variant (SLC8B1 p.Tyr564Ter) was found to co-segregate with disease in a small family with two members diagnosed with MS (Fig 2). This nonsense variant was not observed in any additional samples from MS patients or healthy controls (Table 1).

*SLC8B1* encodes NCLX (also known as SLC24A6), a mitochondrial transporter that mediates calcium extrusion in exchange for either sodium or lithium ions [136]. This mitochondrial inner membrane exchanger provides calcium to the endoplasmic and sarcoplasmic reticulum, and plays a key role in cellular and mitochondrial calcium homeostasis [137]. The SLC8B1 p.Ser94Gly substitution identified in four patients diagnosed with MS is located in the extracellular N-terminal domain, adjacent to the first of thirteen transmembrane segments. In contrast to the variants identified in the other 11 multi-incident families, this serine to glycine substitution is not predicted detrimental to protein function (CADD = 0.005), or evolutionarily conserved (Fig 4). SLC8B1 p.Tyr564Ter, which is predicted to be damaging to protein function (CADD = 39.0), is located in the thirteenth transmembrane domain of SLC8B1, and produces a protein lacking most of the last transmembrane segment, and the entire cytoplasmic C-terminal domain.

Although a direct link between SLC8B1 and the biological processes implicated in the onset of MS has not been described, efflux of mitochondrial calcium has been shown to promote the aggregation of the inflammasome, and to regulate the activation of calcium dependent signaling pathways; including the NFATC-mediated non-canonical Wnt pathway (Fig 3) [138–140]. In addition, SLC8B1 is essential for B-cell motility and chemotaxis, astrocyte function, and synaptic transmission [140–142].

## Discussion

The existence of Mendelian forms of MS has been a recurrent topic of controversy, despite the evidence for familial aggregation, and the measurable increased disease risk for blood-relatives of MS patients [7, 143]. In this study we present the genetic characterization of 34 multi-incident MS families, which have nominated pathogenic variants in 12 genes. Therefore, our data support the existence of Mendelian forms of MS, which can be attributed to a single rare variant of major effect that is largely responsible for the onset of MS and its transmission across generations. However, it should be noted that replication of our findings is warranted as the extremely low MAF observed for these variants, and the relatively low number of carriers within families, precludes sufficient statistical power for meaningful linkage and association analysis.

A monogenic cause for MS could not be identified for 22 families. This was not an unexpected outcome given that complex diseases frequently are genetically heterogeneous, even within families [144, 145]. In these families, pathogenic variants might have been overlooked given that WES technologies are not only unable to assess variants in non-coding regulatory regions, but also do not efficiently capture and sequence all coding exons, and are largely unsuited for the identification of copy number variations and rearrangements which may be responsible for the onset of disease [146]. It is also plausible that our reduced penetrance and phenocopy frequency thresholds are overly stringent, resulting in the exclusion of disease-relevant variants.

The genes harboring rare disease-causing variants for familial MS, herein or previously described [9–12], play critical roles in cellular cation homeostasis, and the regulation of transcription and activation of inflammatory mediators; suggesting a disruption of the innate immune system as the common underlying biological mechanism for the initiation of MS symptoms (Fig 3). Variants in *PLAU*, *MASP1*, and *C2*, as well as risk alleles in *PLG* and *PLAU* [6, 20], suggests a disruption in the fibrinolysis and complement cascade in response to microbial threads or cellular debris as a trigger for MS. In addition, PLAU activation increases angiogenesis, which has been associated with MS severity, and sustains the inflammatory response by providing oxygen and nutrients to the sites of inflammation [147, 148]. Complement genes are necessary for the generation of anaphylatoxins C3a and C5a, opsonisation of pathogens, and formation of the membrane attack complex [39]. Inhibition of the complement system has been shown to reduce the expression of inflammatory mediators, and promote the activation of anti-inflammatory pathways, including the LXR and PPAR nuclear receptor pathways, halting neuroinflammation in the chronic relapsing EAE model [149]. Anaphylatoxins C3a and C5a bind to their corresponding membrane-bound receptors (C3aR, C5aR1 and C5aR2) on the surface of monocytes and macrophages regulating the aggregation of the inflammasome (Fig 3). This complex regulatory mechanism activates or inhibits inflammasome formation in distinct cell types [150, 151], and is mediated through the mobilization of calcium and potassium cations from the extracellular space and intracellular stores to the cytoplasm [26]. Activation of C5aR directly promote the influx of extracellular calcium and release from intracellular stores, whereas activation of C3aR triggers the efflux of intracellular ATP which activates purinergic receptors that mediate calcium influx and potassium efflux through the plasma membrane [152–154]. These include purinergic receptors P2RX4 and P2RX7 in which digenic mutations for familial MS and risk alleles have been described [10, 118]. Regulation of the inflammasome has also been observed in response to increased intracellular calcium concentrations due to sublytic deposition of the membrane attack complex, or decreased expression of NLRP3 and increased expression of NLRP12 in response to C1q (complement component 1, q subcomponent); indicating that numerous elements of the fibrinolysis and complement cascades are capable of regulating the inflammatory response (Fig 3) [150, 155]. A disruption of cellular cation homeostasis in the pathophysiology of MS is further supported by disease-causing variants for multi-incident MS families in potassium channel *KCNG4* and cation exchangers *SLC24A1* and *SLC8B1* (Fig 2).

The activation of the inflammasome has been proposed as a mechanism of autoimmunity in MS patients [156], a hypothesis that is supported by rare variants in inflammasome components *NLRP1*, *NLRP3*, *NLRP5* and *NLRP9* which were identified in MS families, or found to correlate with disease course and severity [8, 11, 54, 56]. In this study we describe two missense substitutions in *NLRP12*, a NOD-like receptor family member that negatively regulates inflammation and NF-κB signaling, while promoting T-cell activation and differentiation [53]. Mutations in *NLRP12* that cause increased secretion of IL-1β have been described in patients with FCAS, and supports a role for NLRP12 in the onset of autoimmune diseases [52]. Interestingly, the NLRP12 p.Leu972His substitution identified in MS patients seems to have the opposite effect (Fig 5C), which may explain why the p.Arg352Cys substitution associated with FCAS had similar frequencies in MS patients and controls, and failed to co-segregate with disease in families.

Activation of purinergic receptors and increased cytosolic calcium concentrations also regulate Wnt signaling pathways by inhibiting glycogen synthase kinase-3-β (GSK3β) and activating the phosphatase activity of calcineurin [90, 91, 157]. GSK3β inhibits both the canonical and non-canonical Wnt signaling pathways by phosphorylating β-catenin and NFAT, thus promoting their nuclear export and degradation [67, 87, 158]. Interestingly, inhibition of GSK3β has been shown to accelerate myelin debris clearance and axonal remyelination [159]. Calcium-bound calcineurin dephosphorylates NFAT which translocates to the nucleus and activates the transcription of the Wnt/Ca^2+^ target genes, including several cytokines, chemokines, and PLAU (Fig 3) [160]. Activated calcineurin also blocks NF-κB and MAPK pathways, inhibiting toll-like receptor signaling in response to pathogens and cellular damage, thus providing another means of innate immune regulation [161]. NFATC2, is one of five NFAT transcription factors, and one of the four genes in the Wnt signaling pathway found to harbor disease-causing variants for familial MS (Fig 2).

In the Wnt/Ca^2+^ pathway, we also identified rare missense variants in *RNF213*, which targets NFATC2 for proteasomal degradation [97]. As previously described, mutations in *RNF213* are associated with MMD, a progressive cerebral angiopathy which may lead to cerebral infarction, but also quasi-MMD which encompasses various clinical entities including autoimmune disease and atherosclerosis [100]. *RNF213* mutations cause these phenotypes through the disruption of cerebral blood flow and reduction of angiogenesis [162]. It is unclear whether KCNG4 and SLC24A1 also play a key role in the activation of the Wnt/Ca^2+^ signaling pathway; however, rapid calcium influx via plasma membrane channels, which is buffered by mitochondrial calcium uptake and slow release through SLC8B1, has been proposed as the mechanism for sustained activation of NFATC2 [139, 163]. Interestingly, mitochondrial dysfunction and impaired calcium sequestration amplify NLRP3 inflammasome signaling [164, 165].

In the canonical Wnt/β-catenin pathway, which is upregulated in response to demyelinating events [159], we identified pathogenic variants in *UBR2* and *CTNNA3* (Fig 2). The activation of the canonical Wnt pathway modulates the immune response by initiating a pro-inflammatory signaling cascade, which includes several cytokines and chemokines, and complement and inflammasome components (Fig 3) [68, 69]. UBR2 has been shown to regulate the activation of the canonical Wnt pathway upstream of β-catenin; and although its mechanism of action still remains to be resolved, depletion of UBR2 leads to reduced expression of β-catenin target genes [78]. In contrast, CTNNA3 as well as CTNNA1 and CTNNA2, the other two members of the α-catenin protein family (Fig 4), inhibit the Wnt/β-catenin pathway [81]. These proteins also play an important role in cell-cell adhesion in ependymal cells, and thus variants identified in MS families could not only disrupt the Wnt signaling pathway, but also BBB integrity [72, 80].

In oligodendrocyte and glial cells, the expression of major components of the canonical Wnt signaling pathway, including β-catenin, is regulated by oxysterols and LXRs [107, 166]. Oxysterols, which can modulate the innate and adaptive immune response, bind LXRs activating the nuclear receptor complex and promoting the initiation of transcription [167]. Genetic association and familial mutations for MS have been described in components of the oxysterol synthesis pathways and nuclear receptor complex [6, 9, 168, 169]. In addition, a missense variant in nuclear receptor co-activator *NCOA3*, causing increased expression of inflammatory mediators in microglial cells (Fig 5D), was identified in three multi-incident MS families (Fig 2). Although NCOA3 is considered a co-activator that directly binds to nuclear receptors promoting transcriptional activities, it can also serve as a co-activator for NF-κB, enhancing the expression of target genes and maintaining the immune response [170, 171]. Moreover, molecules acting as NR1H3 (LXR-α) agonists have been shown to inhibit NLRP3 inflammasome by downregulating the expression of its components, and NF-κB signaling by suppressing the phosphorylation of IκB (Fig 3) [172]; thus providing additional links between these pro-inflammatory pathways. The identification of rare variants co-segregating with disease in families and genetic associations in components of the nuclear receptor complex and oxysterol synthesis pathways, suggest an important role for these genes in the pathophysiology of MS by regulating not only the synthesis of inflammatory mediators, but also neuronal development, oligodendrocyte differentiation and myelin synthesis [114, 115, 166, 173, 174].

Although replication of our findings in additional multi-incident MS families is necessary to confirm a pathogenic role for these genes and rare variants, they suggest disruption of innate immunity, inflammation, angiogenesis and cation homeostasis as critical processes in the onset of Mendelian forms of MS. Although these genes provide a mechanistic insight into the etiology of disease, it should be noted that not all family members harboring the nominated disease-causing variants developed MS (Fig 2). Therefore, despite the highly susceptible genetic background created by these variants, additional genetic, epigenetic or environmental factors are likely required to trigger the onset of MS.

Although the variants identified in these families are rare, they provide the means for the development of cellular and animal models based on human genetic etiology. Models in which to further characterize the biological pathways disrupted in MS patients, and develop and assess the efficacy of novel therapeutic options tackling the pathophysiological processes of MS. In addition, we envision gene screening being used as a tool for disease confirmation, and accurate risk assessment in healthy family members of MS patients. Following the confirmation of pathogenicity in additional MS families, and with the knowledge gained from characterizing newly developed models of MS based on the identified variants, we foresee the development of personalized treatments for MS patients, and preventative strategies for at risk individuals. These may include PPAR and LXR agonists for MS patients and unaffected family members harboring substitutions in *NCOA3*, and calcium channel blockers for those with variants in *SLC24A1* or *SLC8B1*.

In conclusion, the implementation of WES in multi-incident MS families have nominated pathogenic variants in 12 genes, which highlight innate immunity and inflammatory response as critical processes leading to the onset of MS. A global effort towards the analysis of additional MS families, and the characterization of the biological processes disrupted by these variants, is necessary to expand our knowledge and understanding of the molecular and biological mechanisms underlying the genesis of MS. This gained knowledge is essential to drive the development of personalized medicine approaches with the potential to improve treatment efficacy and patient prognosis.

## Methods

### Participants

A total of 33 multi-incident MS families of European descent from Canada and 1 from Germany were selected for this study. DNA was available for 191 family members diagnosed with MS, 423 unaffected family members and 48 married-in individuals. In each family, DNA was available for at least 4 MS patients (mean = 5.46, SD ± 1.58, range = 4–11). Additional samples from Canada, and Italy were available for the replication of all nominated variants. NCOA3 p.Arg485Cys was additionally genotyped in cohorts from Spain and Austria. The Canadian cohort was collected through the longitudinal Canadian Collaborative Project on the Genetic Susceptibility to Multiple Sclerosis (CCPGSMS), and consists of 13,870 samples (2,502 MS probands which include 2,039 with a family history of MS, 2,390 additional family members diagnosed with MS, 7,903 family members free of MS symptoms, and 1,075 unrelated healthy controls) [175, 176]. The male to female ratio for MS probands and unrelated controls was 1:2.76 and 1:0.96, respectively; and with a mean age at onset for MS patients of 30.8 years (SD ± 9.6). The Spanish cohort consisted of 3,200 MS patients and 2,803 healthy controls, with a male to female ratio of 1:1.88 and 1:1.49, respectively, and a mean age at MS onset of 30.7 years (SD ± 11.7). The Austrian cohort consisted of 552 MS patients with a male to female ratio of 1:2.4 and a mean age at MS onset of 31.4 years (SD ± 9.7). The Italian cohort included 46 MS patients and 32 healthy relatives from 15 multi-incident families recruited as part of the InTegrative Analysis of famiLies with MultIple Sclerosis of ItaliAN Origins (ITALIANO) multicenter study.

The large majority of CCPGSMS probands self-report European descent (98.0%), and the remainder reported Asian ancestry (1.6%), African ancestry (0.3%) or First Nations (0.1%). Samples from European cohorts are of Caucasian ancestry. All patients were diagnosed with MS according to Poser or McDonald criteria [177, 178]. The ethical review boards at each institution approved the study [University of British Columbia ethical review board (H08-01669); Medical University of Vienna ethics committee (EK Nr:2195/2016); San Raffaele Ethical Committee (NEUFAM); Comité Ético de Investigación de Euskadi (CEIC_E300911); Fondo de Investigaciones Sanitarias, Instituto de Salud Carlos III—Fondo Europeo de Desarrollo Regional (FIS PI13/00879 and PI16/01259); Hospital Regional Universitario de Málaga (CTS7670/11, sample collection: C-36-003); and Hospital Virgen Macarena de Sevilla (PI13/01527 and 2254)], and all participants provided written informed consent.

### Sequencing and genotyping analysis

WES data for Canadian samples was generated on an Ion Torrent Proton (Thermo Fisher Scientific) system with a 100× minimum average sequencing depth. The Ion Torrent Server v4 was used to map reads to NCBI Build 37.1 reference genome using the Torrent Mapping Alignment Program (TMAP) and to identify variants differing from the reference. Sequences with a mapping Phred quality score under 20, fewer than five reads or over 95% strand bias were excluded from further analysis [9, 169]. German and Italian samples were sequenced on a HiSeq 2500 (Illumina), and the raw sequences were aligned against the human reference genome (hg19) with BWA and processed with a GATK best practices pipeline using Unified Genotyper variant caller. Annotation of variants was performed with ANNOVAR [179].

WES data for 132 MS patients from 34 families was generated for the identification of pathogenic variants (S4 Table). Heterozygote non-silent variants identified in WES data from all patients in a single family, and with a MAF below 1% in public (ExAC) or proprietary databases of variants [17], were genotyped in all family members to validate WES genotype calls and assess segregation with disease, and Canadian MS probands and healthy controls to assess population frequencies, as previously described [9]. To account for reduced penetrance and the presence of phenocopies, variants were deemed to segregate with disease when found in at least 75% of individuals diagnosed with MS and no more than one unaffected family member, excluding unaffected parents of MS patients. When a variant segregating with disease could not be found, additional affected family members for whom DNA was available were analyzed by WES, and rare non-silent variants identified in all but one MS patient were assessed for segregation with disease. Additional variants in each gene of interest were identified by mining WES data from 426 MS patients from Canada, 15 probands from multi-incident MS families from Italy, 100 healthy controls from Canada, and 955 multi-ethnic diseased controls. Missense or nonsense variants identified exclusively in MS patients, and with a MAF below 1% in public databases of variants [17] were assessed for segregation within families.

All variants deemed to co-segregate with disease were genotyped using Sequenom MassArray iPLEX platform or TaqMan genotyping probes (Tables 1 & S5). For every additional patient identified harboring a variant of interest, all blood-related family members for whom DNA was available were genotyped using Sanger sequencing to confirm genotype calls and assess segregation with disease as previously described [169, 180].

Haplotype analysis were performed using microsatellite markers spanning each locus of interest. Primer sequences are available at the National Centre for Biotechnology and Information (https://www.ncbi.nlm.nih.gov/probe). PCR reactions were performed under standard conditions with one primer pair for each marker labeled with a fluorescent tag. PCR products were run on an ABI 3730xl (Applied Biosystems) and analyzed using GeneMapper 4.0. Marker sizes were normalized to those reported in the Centre d’Etude du Polymorphisme Humain (CEPH) database (http://www.cephb.fr/).

### Construct design, western blotting and luciferase analysis

Plasmids containing FLAG-tagged full length wild-type, p.Gln475 or p.His972 human NLRP12 were kindly donated by Dr. Beckley Davis (Franklin & Marshall College, USA). Full length cDNA encoding wild-type NCOA3 was PCR amplified from total human brain cDNA, and the p.Arg485Cys substitution introduced by fusion PCR. After restriction digestion, PCR products were inserted into pcDNA4-myc-his A (pZ) between *Kpn*I and *Xho*I.

1–2μg of empty vector or expression vectors for NLRP12 wild-type (WT), NLRP12 p.Gln475 (L475Q), NLRP12 p.His972 (L972H), NCOA3 wild-type (WT), or NCOA3 p.Cys485 (R485C) was transfected into mouse microglial cell line BV2 using polyethylenimine (PEI). Twenty-four hours after transfection, whole cell lysates were subjected to Western blot for Caspase-1/p10 (Santa Cruz, Cat# sc-56036, RRID: AB_781816), FLAG-NLRP12 (Sigma, Cat# F7425, RRID: AB_439687) and β-actin (Sigma, Cat# A5316, RRID: AB_476743), or iNOS (Cell Signaling Technology, Cat# 13120, RRID: AB_2687529), NCOA3 (Cell Signaling Technology, Cat# 2126S, RRID: AB_823642) and β-actin. Activation of NF-κB was assessed in HEK293 cells transfected with 200ng of a pcDNA4 vector containing the coding region for either wild-type, p.Gln475 or p.His972 NLRP12, together with 200ng of a reporter PGL3 plasmid with a response element for NF-κB. Cells were co-transfected with a vector to express p65 in order to induce NF-κB activation. 15ng of a vector containing Renilla Luciferase was transfected into cells as an internal control. Twenty-four hours post-transfection cells were lysed, and luciferase activity in cell lysates was measured using a luciferase assay kit (Promega, Cat# E1500). All experiments were performed at least in triplicate, and protein bands quantified with Quantity One (Bio-Rad). One-way ANOVA and Tukey’s Honest Significant Difference (HSD) post hoc test were used to identify statistically significant differences between groups.

## Supporting information

S1 Table Clinical phenotype for MS patients harboring mutations in nominated MS genes. RR, relapsing-remitting MS; PP, primary progressive MS; RR (SP), relapsing-remitting which became secondary progressive; n/a, not available. *Disease course was determined post hoc from clinical charts.
(PDF)

S2 Table Non-segregating variants identified in MS patients. *excluding parents of MS patients.
(PDF)

S3 Table NCOA3 and SLC24A1 disease haplotypes. Microsatellite markers are shown with their physical locations (NCBI Build 37.1, hg19). Allele sizes are given in base pairs consistent with Centre d’Etude du Polymorphisme Humain (CEPH) standards, and include CEPH samples 1331–1 and 1331–2 as reference.
(PDF)

S4 Table WES variants identified in the 34 MS families characterized in this study. Genomic coordinates from NCBI Build 37.1 (hg19) and dbSNP refSNP (rs) identifiers from build 147 are provided. Minor allele frequency (MAF) for MS families and the Genome Aggregation Database (GnomAD) are given.
(ZIP)

S5 Table Allele frequencies in Canadian case-control samples for additional variants segregating with disease in MS families. Genomic coordinates from NCBI Build 37.1 (hg19) and minor allele frequencies (MAF) are provided.
(PDF)

S1 Fig Segregation analysis and conservation for a) MASP1 p.Pro462Thr and b) RNF213 p.Arg4019Cys. Males are represented by squares and females by circles, a diagonal line indicates subjects known to be deceased. Black filled symbol, MS; gray filled, unaffected obligate carrier. Heterozygote carriers (M) and wild-type (wt) genotypes are provided. MS patients with inferred genotypes are indicated with an asterisk. Organism and RefSeq accession numbers are provided for orthologs and gene name and RefSeq accession numbers for human paralogs, which were obtained from Ensembl release 91. Evolutionarily conserved positions for nominated pathogenic variants are highlighted in black.
(PDF)

S2 Fig NLRP12 inhibition of NF-κB pathways. Relative NF-κB promoter activity ± standard error for wild-type and mutant NLRP12 constructs is provided; n.s., not significant.
(PDF)
