# Expression of Concern: Exome sequencing in multiple sclerosis families identifies 12 candidate genes and nominates biological pathways for the genesis of disease

**DOI:** 10.1371/journal.pgen.1008436

**Published:** 2019-10-11

**Authors:** The PLOS Genetics Editors

The editors of *PLOS Genetics* are issuing this expression of concern to alert readers to questions about the validity of some statements in the article authored by Carles Vilariño-Güell and colleagues [1].

Shortly after the article was published, readers contacted the journal with concerns regarding the soundness of the research described and the rigor of the editorial process. Internal investigation of the review and editorial process of the manuscript revealed no evidence of scientific misconduct or conflict of interest. However, it was noted by the editors involved in the investigation that there were important discrepancies between the actual results and the claims that were made in parts of the manuscript. Because those discrepancies had the potential to introduce errors in clinical genetic test interpretation and cause harm, the journal contacted the authors to request that they consider issuing corrections to two sentences in the Abstract, two sentences in the Author summary, and one sentence in the Discussion.

Responding on behalf of the authors, Carles Vilariño-Güell, the corresponding author, did not agree to the requested corrections. In the editors’ opinion these corrections were required to prevent potential harm. The authors and their institution have been made aware of this Expression of Concern and the journal has initiated a more extensive examination of the review and editorial process. The outcome of these inquiries will determine whether further action is required. The editors involved in issuing this Expression of Concern have also authored an Editorial [2] providing additional information.
