# Correction: Exome sequencing in multiple sclerosis families identifies 12 candidate genes and nominates biological pathways for the genesis of disease

**DOI:** 10.1371/journal.pgen.1008737

**Published:** 2020-04-09

**Authors:** The PLOS Genetics Editors

After publication of this article [1], concerns were raised about the validity of some statements in the article. In light of these, an Expression of Concern was issued by the *PLOS Genetics* Editors on October 11, 2019 [2]. Since that time, the Editors have reassessed the article in collaboration with additional experts in the field. Based on the outcome of this assessment and advice received, the Editors concluded that causal statements regarding the relationship between specific DNA variants and multiple sclerosis (MS) are not adequately supported. Community standards established by the American College of Medical Genetics and Genomics and the Association for Molecular Pathology [3] advise that the term "likely pathogenic" should be reserved for situations when evidence that a specific variant causes a disease meets defined criteria, and the strength of such evidence is sufficiently high to merit providing the information to patients or genetic test recipients so as to inform clinical decision-making. The assessment of the Editors, supported by the consensus of experts consulted in this case, is that statements in the article suggesting a causal relationship between the genetic variants and MS do not abide by this guidance, and they introduce risk of clinical harm through their potential to mislead patients and health care providers, and/or to influence clinical decision-making and the interpretation of genomic testing.

In addition, concerns were raised in this assessment that the Abstract does not adequately acknowledge the importance of replication in supporting the article’s findings.

Based on the outcome of the post-publication assessment, and to address the above concerns, we make the following updates to the article’s text:

1. In the third sentence of the Abstract, the phrase "…which nominated likely pathogenic variants for MS in 12 genes…" should read, “…which identified variants that co-segregated with MS in 12 families, and represented 12 genes…”
2. The last sentence of the Abstract should be replaced with the following text: “The relatively low number of carriers within MS families, together with the very low minor allele frequency observed for most of the 12 variants, preclude meaningful linkage and/or association analysis, and it will be important to replicate our findings. Nonetheless, the genes we identified suggest a disruption of interconnected immunological and pro-inflammatory pathways as the initial event in the pathophysiology of familial MS, and provide the molecular and biological rationale for the chronic inflammation, demyelination and neurodegeneration observed in MS patients.”
3. The following sentence in the Author summary should be removed: “In these families, the cause of multiple sclerosis can be largely attributed to a single genetic variant that is transmitted through generations.”
4. In the third sentence of the Author summary, the phrase " … 12 rare genetic variants that are largely responsible for the onset of multiple sclerosis in these families," should read, “…12 rare genetic variants that are often transmitted together with multiple sclerosis in these families.”
5. The second sentence of the Discussion: “In this study we present the genetic characterization of 34 multi-incident MS families, which have nominated pathogenic variants in 12 genes,” should read, “In this study we present the genetic characterization of 34 multi-incident MS families, which have identified variants in 12 genes that co-segregated with MS.”
6. In the last paragraph in the Discussion, the first sentence: “In conclusion, the implementation of WES in multi-incident MS families have nominated pathogenic variants in 12 genes, which highlight innate immunity and inflammatory response as critical processes leading to the onset of MS,” should read, “In conclusion, the implementation of WES in multi-incident MS families have identified variants in 12 genes which may be involved in processes leading to the onset of MS.”

Similarly, other statements in the article inferring that the article’s results support a causal link between a variant and MS should be interpreted as referring to correlative rather than causal associations. This applies, for example, to statements in the Discussion referring to "disease-causing" or "pathogenic" variants, and to sentences that draw conclusions about the roles of pathways/processes involving the genes of interest in the etiology of MS.

These corrections resolve the prior Expression of Concern.

The corresponding author confirmed that the authors as a group support this Correction.

FM-B, KV, EM, AnZ, FE, JM, AlA, and ADS agreed to the contents of this Correction notice.

CV-G, AlZ, BH, ZW, FM, EU, LL, DG, CM, JAQ, ALT, ME, CQB, JF, IMY, MGC, AD, EMR, TZ, AnA, GI, LE-P, PU-R, JO-P and WS did not agree to all of the above corrections.
