# Supplementary material for: Exome sequencing in multiple sclerosis families identifies 12 candidate genes and nominates biological pathways for the genesis of disease
Source: PLoS Genet. 2019 Jun 6;15(6):e1008180. doi: 10.1371/journal.pgen.1008180 (PMC6553700; doi:10.1371/journal.pgen.1008180)
Supplement: S1 Table — RR, relapsing-remitting MS; PP, primary progressive MS; RR (SP), relapsing-remitting which became secondary progressive; n/a, not available. *Disease course was determined post hoc from clinical charts. (PDF) [file pgen.1008180.s001.pdf]

Table S1 - Clinical phenotype for MS patients harboring variants in nominated MS genes.
RR, relapsing-remitting MS; PP, primary progressive MS; RR (SP), relapsing-remitting which became secondary progressive; n/a, not available. *Disease course was determined post hoc from clinical charts.

Gene and amino acid change	Patient ID	Gender	Age at onset (age at death)	Disease course at MS onset	EDSS (at years after MS onset)
PLAU p.C151F	II-1	Female	n/a (52)		10 (n/a)
	II-4	Male	34		
	II-5	Male	23 (44)	RR*	10 (21)
	II-6	Female	36	RR	2 (0)
PLAU p.T86A	II-1	Male	27	PP	6.5 (11)
	II-2	Female	17	RR	1 (19)
PLAU p.H149Y	II-6	Female	25	PP	8 (25)
	III-2	Female	25	RR	3.5 (3)
MASP1 p.G459D	II-1	Female	20	RR (SP)	6 (26)
	II-2	Female	33	RR	3 (28)
	II-3	Male	28	RR	3 (34)
	II-5	Female	22	RR (SP)	4 (22)
	II-8	Male	34	RR (SP)	6.5 (35)
C2 p.T184M	III-2	Female	31 (47)	RR*	10 (16)
	IV-1	Male	31	RR	
	IV-6	Female	38	RR	2.5 (7)
	IV-7	Female		RR (SP)	8 (n/a)
NLRP12 p.L972H	II-1	Male	23 (44)	PP	3.5 (23)
	II-2	Female	25	PP	7.5 (19)
	II-4	Female	32	RR	3.5 (26)
	II-5	Female	32	RR (SP)	4 (11)
	II-6	Male	35		
	II-7	Female			
NLRP12 p.L475Q	II-1	Male	39	PP	7.5 (4)
	II-2	Female	22	RR	0 (14)
UBR2 p.A1658T	II-3	Female	47	PP	8 (16)
	II-7	Male	36	RR	6 (17)
	III-2	Female	38	PP	6 (14)
	III-4	Male	29	PP	8 (5)
CTNNA3 p.A852S	III-3	Female	39	RR	
	III-6	Female	30	RR	
	III-10	Female	25	PP	3.5 (25)
	III-11	Male	24	RR	1.5 (9)
	III-12	Female	15	PP	7 (24)
NFATC2 p.P679L	III-2	Female	23	PP	
	III-4	Female	30	PP	6.5 (44)
	III-9	Female			
	III-10	Male			
	IV-3	Female	42	RR	1.5 (5)
	IV-4	Female	37	RR	1.5 (1)

Table S1 - (Continued)

Gene and amino acid change	Patient ID	Gender	Age at onset (age at death)	Disease course at MS onset	EDSS (at years after MS onset)
RNF213 p.N2327D	II-1	Female	37	RR	0 (8)
	II-3	Female	30	RR	0 (12)
	II-5	Female	24 (44)	RR (SP)	9 (15)
	II-6	Female	29	RR	1.5 (3)
	II-7	Female	36		
NCOA3 p.R485C	III-2	Female	23	PP	
	III-7	Female	37	PP	
	III-8	Female			
	IV-4	Female			
	II-8	Female	23	PP	8 (43)
	III-10	Female	25	RR	1.5 (20)
	II-12	Female	27		
	II-13	Female	32 (56)		
KCNG4 p.R474H	III-2	Male			
	III-4	Male	25	RR	0 (19)
	III-9	Female	14 (54)	PP	9 (34)
	IV-1	Male	22	RR	1.5 (3)
	IV-2	Female	20	RR	
	IV-3	Female	27	RR	
KCNG4 p.R365H	III-3	Female	49	PP	6 (8)
	III-5	Female	37	PP	7 (21)
SLC24A1 p.L26F	II-2	Male	48 (79)	PP	8 (19)
	II-4	Female	41	PP	8 (23)
	II-9	Female	25	RR*	
	III-1	Male	25	PP*	
	III-2	Male	30	RR*	
	III-3	Female	20	RR	1 (1)
	II-10	Male	n/a (78)		
	III-4	Female	40	RR	2 (14)
	II-14	Male	25	RR (SP)	2.5 (10)
SLC8B1 p.S94G	III-2	Male	23	RR (SP)	6.5 (32)
	III-3	Female	22		
	III-5	Female	50		
	III-7	Female	24	RR	6 (22)
SLC8B1 p.Y564X	II-1	Male		PP	
	III-1	Female	22	RR	1.5 (23)
