# Supplementary material for: Exome sequencing in multiple sclerosis families identifies 12 candidate genes and nominates biological pathways for the genesis of disease
Source: PLoS Genet. 2019 Jun 6;15(6):e1008180. doi: 10.1371/journal.pgen.1008180 (PMC6553700; doi:10.1371/journal.pgen.1008180)
Supplement: S2 Table — *excluding parents of MS patients. (PDF) [file pgen.1008180.s002.pdf]

Table S2 - Non-segregating variants identified in MS patients.

Gene	Chromosome: Genomic position	Reference/ Alternative allele	Transcript ID	cDNA change	Amino acid change	dbSNP ID rs/ss number	Population	Number of families	MS: carriers/ total	Healthy: carriers/ total*	ExAC (MAF)
PLAU	10:75671487	A/T	NM_002658	c.412T>A	p.S138T	rs776428822	Italy	2	1/5, 1/3	-	0.009%
MASP1	3:186954337	C/T	NM_139125	c.1322G>A	p.R441H	rs141872329	Canada	1	1/2	0/3	0.03%
	3:186954275	G/T	NM_139125	c.1384C>A	p.P462T	ss2137543902	Canada	1	3/5	1/3	n/a
	3:186954047	G/A	NM_139125	c.1612C>T	p.R538X	rs369868022	Canada	1	1/2	1/1	0.001%
C2	6:31911430	A/G	NM_000063	c.1577A>G	p.K526R	rs146054348	Canada	1	1/10	-	0.02%
NLRP12	19:54313859	G/A	NM_144687	c.1054C>T	p.R352C	rs199881207	Italy/Canada	2	2/4, 1/2	1/2, 1/3	0.04%
UBR2	6:42600308	G/A	NM_015255	c.1300G>A	p.E434K	rs769174334	Canada	1	2/3	2/6	0.002%
	6:42604807	G/A	NM_015255	c.1733G>A	p.G578D	rs780141170	Canada	1	1/4	1/3	0.001%
	6:42643860	G/A	NM_015255	c.4318G>A	p.G1440R	rs371184591	Canada	1	1/2	1/3	0.006%
CTNNA3	10:69366752	G/A	NM_013266	c.155C>T	p.S52L	rs139105272	Italy	2	1/4, -	-, 1/4	0.02%
	10:68979412	G/A	NM_013266	c.796C>T	p.P266S	rs199985402	Canada	1	1/3	1/2	0.007%
	10:68940214	C/T	NM_013266	c.908G>A	p.R303H	rs780671006	Canada	1	1/10	-	0.002%
	10:68526171	G/A	NM_013266	c.1132C>T	p.R378C	rs187752783	Canada	2	1/4, 1/10	2/4, -	0.04%
	10:68280458	T/C	NM_013266	c.1448A>G	p.K483R	rs200282168	Italy	1	1/3	-	n/a
	10:67680203	A/G	NM_013266	c.2573T>C	p.L858S	rs41313840	Canada	2	1/2, 1/4	2/4, -	0.03%
NFATC2	20:50140514	G/T	NM_173091	c.266C>A	p.P89Q	rs556060763	Italy	1	1/4	-	0.007%
	20:50051820	C/T	NM_173091	c.1937G>A	p.R646Q	rs769925564	Canada	1	2/2	5/6	0.005%
	20:50049200	G/A	NM_173091	c.2126C>T	p.P709L	rs751513686	Canada	1	1/7	-	0.002%
	20:50049108	C/T	NM_173091	c.2218G>A	p.A740T	rs758439316	Canada	1	2/5	2/2	0.004%
	20:50049105	G/C	NM_173091	c.2221C>G	p.R741G	rs78964722	Canada	1	1/3	1/3	0.02%
RNF213	17:78313149	C/T	NM_001256071	c.4982C>T	p.T1661M	rs144944826	Italy	1	1/3	-	0.01%
	17:78317719	A/G	NM_001256071	c.6246A>G	p.I2082M	rs778899448	Canada	1	1/2	1/4	0.001%
	17:78317763	T/C	NM_001256071	c.6290T>C	p.V2097A	rs375665425	Canada	1	1/3	0/3	0.001%
	17:78318686	A/G	NM_001256071	c.6551A>G	p.Q2184R	rs138595111	Italy	1	1/3	-	0.24%
	17:78320968	C/T	NM_001256071	c.8833C>T	p.R2945C	rs540094757	Canada	1	1/2	1/2	0.06%

RNF213	17:78321229	G/A	NM_001256071	c.9094G>A	p.G3032R	rs148408469	Canada	1	1/5	0/5	0.001%
	17:78326787	A/T	NM_001256071	c.10351A>T	p.M3451L	rs532730964	Canada	1	1/5	-	0.007%
	17:78327338	G/A	NM_001256071	c.10450G>A	p.G3484S	rs140300282	Canada	1	1/1	2/5	0.05%
	17:78336951	C/G	NM_001256071	c.11405C>G	p.S3802X	rs757791430	Canada	1	1/2	2/5	0.001%
	17:78341843	C/T	NM_001256071	c.12055C>T	p.R4019C	rs139265462	Canada	2	1/4, 2/2	0/3, -	0.05%
	17:78343324	C/T	NM_001256071	c.12178C>T	p.R4060C	rs144719362	Canada	1	1/2	-	0.01%
	17:78345775	A/G	NM_001256071	c.12527A>G	p.N4176S	rs527844265	Italy	2	1/5, -	-, 1/2	0.01%
	17:78353463	C/T	NM_001256071	c.13589C>T	p.A4530V	rs151100315	Canada	1	3/6	-	0.01%
	17:78359398	T/C	NM_001256071	c.14516T>C	p.L4839P	ss3646580240	Canada	1	1/3	2/4	n/a
	17:78360130	C/T	NM_001256071	c.14620C>T	p.R4874W	rs779668238	Canada	1	1/3	-	0.001%
	17:78360187	G/C	NM_001256071	c.14677G>C	p.E4893Q	rs143163385	Canada	1	2/4	1/4	0.04%
NCOA3	20:46262252	A/G	NM_181659	c.836A>G	p.N279S	rs756473801	Canada	1	1/2	2/4	0.003%
	20:46265385	C/T	NM_181659	c.2255C>T	p.S752F	rs61748368	Italy	2	1/5, 1/4	1/4, -	0.04%
	20:46268423	C/G	NM_181659	c.2810C>G	p.S937C	rs142951578	Canada	1	1/2	-	0.05%
	20:46268497	C/T	NM_181659	c.2884C>T	p.P962S	ss3646580243	Canada	1	1/2	2/5	n/a
	20:46275917	A/C	NM_181659	c.3353A>C	p.Q1118P	rs149561356	Italy	2	2/5, -	1/4, 1/2	0.27%
	20:46275938	G/A	NM_181659	c.3374G>A	p.G1125D	rs144254604	Canada	2	3/4, 1/2	2/5, 1/4	0.03%
	20:46279918	C/T	NM_181659	c.3844C>T	p.P1282S	rs138733364	Italy	1	1/3	1/2	0.14%
KCNG4	16:84270469	C/T	NM_172347	c.623G>A	p.R208H	rs560844482	Canada	1	1/6	-	0.001%
	16:84256355	C/T	NM_172347	c.1028G>A	p.R343H	rs377698250	Canada	1	3/4	3/6	0.02%
SLC24A1	15:65916899	C/T	NM_004727	c.481C>T	p.Q161X	rs200047855	Canada	1	1/7	-	0.003%
	15:65917553	A/T	NM_004727	c.1135A>T	p.T379S	rs374623693	Canada	1	1/10	-	0.002%
	15:65918247	T/G	NM_004727	c.1829T>G	p.V610G	ss3646580238	Canada	1	1/3	3/8	n/a
	15:65944027	T/G	NM_004727	c.2813T>G	p.V938G	rs376493994	Canada	1	2/4	2/4	0.001%
	15:65944981	T/C	NM_004727	c.2885T>C	p.V962A	rs751019241	Canada	1	2/4	4/6	0.003%
SLC8B1	12:113758446	T/C	NM_024959	c.466A>G	p.S156G	ss3646580237	Canada	1	2/2	3/5	n/a
	12:113754399	G/A	NM_024959	c.925C>T	p.L309F	rs758828313	Canada	1	2/5	2/3	0.002%
	12:113753176	G/A	NM_024959	c.1099C>T	p.P367S	rs763829480	Canada	1	1/10	-	0.005%
	12:113748055	G/A	NM_024959	c.1241C>T	p.P414L	ss3646580236	Italy	1	2/3	1/2	n/a

*excluding unaffected parents of MS patients
