# Supplementary material for: Exome sequencing in multiple sclerosis families identifies 12 candidate genes and nominates biological pathways for the genesis of disease
Source: PLoS Genet. 2019 Jun 6;15(6):e1008180. doi: 10.1371/journal.pgen.1008180 (PMC6553700; doi:10.1371/journal.pgen.1008180)
Supplement: S3 Table — Microsatellite markers are shown with their physical locations (NCBI Build 37.1, hg19). Allele sizes are given in base pairs consistent with Centre d’Etude du Polymorphisme Humain (CEPH) standards, and include CEPH samples 1331–1 and 1331–2 as reference. (PDF) [file pgen.1008180.s003.pdf]

Table S3 - NCOA3 and SLC24A1 disease haplotypes.

Microsatellite markers are shown with their physical locations (NCBI Build 37.1, hg19). Allele sizes are given in base pairs consistent with Centre d'Etude du Polymorphisme Humain (CEPH) standards, and include CEPH samples 1331-1 and 1331-2 as reference.

a)

Marker	Chromosome and position	NCOA3 Family 1	NCOA3 Family 2	NCOA3 Family 3	CEPH 1331-1	CEPH 1331-2
D20S888	20:45,183,737	159	161	161	155/159	147/163
D20S886	20:45,251,083	151	145	147	145/151	147/147
D20S891	20:45,929,588	216	212	204	212/212	204/210
D20S197	20:46,160,704	191	191	191	191/205	187/191
NCOA3 R485C	20:46,264,406	T	T	T	C	C
D20S178	20:46,551,927	254	248	248	248/250	244/254
D20S176	20:47,313,442	173	167	173	167/185	173/173
D20S866	20:47,363,334	179	183	173	171/173	171/173
D20S887	20:47,673,170	261	245	245	253/255	245/245

b)

Marker	Chromosome and position	SLC24A1 Family 1	SLC24A1 Family 2	SLC24A1 Family 3	CEPH 1331-1	CEPH 1331-2
D15S987	15:63,298,398	176	176	182	176/177	176/176
D15S159	15:63,426,872	163	165	163	163/169	165/165
D15S993	15:64,217,748	181	181	187	179/183	181/183
D15S1507	15:65,340,003	204	208	216	204/208	208/212
SLC24A1 L26F	15:65,916,494	T	T	T	C	C
D15S1020	15:65,998,580	218	218	218/227	221/227	218/221
D15S213	15:66,452,868	190	192	190	190/192	190/192
D15S153	15:66,559,862	218	208	208	212/218	212/218
D15S125	15:67,092,827	159	173	173	167/169	165/165
D15S988	15:67,326,520	277	275	263	261/267	259/261
