# Supplementary material for: Exome sequencing in multiple sclerosis families identifies 12 candidate genes and nominates biological pathways for the genesis of disease
Source: PLoS Genet. 2019 Jun 6;15(6):e1008180. doi: 10.1371/journal.pgen.1008180 (PMC6553700; doi:10.1371/journal.pgen.1008180)
Supplement: S5 Table — Genomic coordinates from NCBI Build 37.1 (hg19) and minor allele frequencies (MAF) are provided. (PDF) [file pgen.1008180.s005.pdf]

Table S5 - Allele frequencies in Canadian case-control samples for additional variants segregating with disease in MS families.

Chromosome	Position (hg19)	Reference	Alternate	MS (MAF)	Control (MAF)
1	1,225,899	G	A	1.275%	1.056%
1	1,232,004	G	A	0.528%	0.507%
1	9,656,015	A	T	0.020%	0%
1	12,342,990	G	A	1.037%	1.046%
1	12,835,153	G	A	0.040%	0.047%
1	60,331,604	T	C	0.020%	0%
1	109,824,441	G	C	0.044%	0%
1	186,957,518	G	A	0.111%	0.111%
2	25,966,789	A	C	0.024%	0%
2	44,539,792	T	C	0.366%	0.185%
2	53,943,868	A	G	0.369%	0.252%
2	109,365,537	A	G	0.025%	0%
2	109,546,682	G	A	0.073%	0%
2	160,994,593	T	C	0.024%	0%
2	163,361,158	T	A	0.593%	0.411%
2	201,436,400	G	T	0.020%	0%
2	210,569,316	G	A	0.020%	0%
3	69,239,047	G	T	0.199%	0.169%
4	100,261,822	T	A	0.040%	0%
5	89,979,568	G	A	0.507%	0.358%
5	100,147,570	C	T	0.024%	0%
5	150,901,488	T	C	0.049%	0%
5	162,945,202	A	G	0.020%	0%
6	20,124,710	G	A	0.243%	0.050%
6	26,124,044	C	T	0.020%	0%
6	26,271,372	T	C	0.049%	0%
6	27,925,237	C	G	0.020%	0%
6	32,820,000	C	A	0.573%	0.462%
6	33,143,391	G	A	0.100%	0.047%
6	35,196,417	A	G	0.020%	0%
6	40,999,437	T	C	0.398%	0.508%
6	42,656,072	G	A	0.020%	0%
6	49,814,332	C	T	0.049%	0.061%
6	109,768,568	G	T	0.557%	0.333%
6	137,166,790	A	C	0.534%	0.825%
6	150,157,303	C	A	0.030%	0.059%
7	47,342,634	G	A	0.025%	0%
7	48,626,771	C	T	0.391%	0.366%
9	131,337,087	A	G	0.025%	0%
11	397,045	C	T	0.223%	0.050%
11	9,769,601	G	C	0.020%	0%
11	45,672,298	C	T	0.024%	0%

Table S5 - (Continued)

Chromosome	Position (hg19)	Reference	Alternate	MS (MAF)	Control (MAF)
11	62,297,555	C	T	0.119%	0.118%
11	67,223,136	C	T	0.122%	0%
11	86,055,720	A	G	0.025%	0%
14	21,458,649	C	G	1.111%	1.346%
14	101,201,147	G	A	0.040%	0%
15	28,519,518	A	G	0.020%	0.049%
15	43,571,404	C	T	0.807%	0.507%
16	2,229,597	G	C	0.040%	0.049%
16	5,009,353	G	A	0.020%	0%
17	80,543,936	C	T	0.628%	0.596%
18	10,705,481	T	C	0.073%	0.062%
18	12,794,360	G	T	0.025%	0%
18	42,531,274	G	A	0.024%	0.062%
19	39,230,735	C	T	0.072%	0.243%
19	39,876,646	A	G	0.719%	1.065%
19	41,117,300	G	A	1.030%	1.055%
19	54,600,361	C	T	0.071%	0%
20	2,464,183	C	T	0.024%	0.059%
20	23,546,723	A	G	0.044%	0.057%
20	40,053,863	C	T	0.177%	0.113%
20	43,945,472	C	G	0.044%	0.057%
20	45,130,768	G	C	0.221%	0.226%
22	25,124,311	G	A	0.133%	0.056%
