# Supplementary material for: Exome sequencing in multiple sclerosis families identifies 12 candidate genes and nominates biological pathways for the genesis of disease
Source: PLoS Genet. 2019 Jun 6;15(6):e1008180. doi: 10.1371/journal.pgen.1008180 (PMC6553700; doi:10.1371/journal.pgen.1008180)
Supplement: S1 Fig — Males are represented by squares and females by circles, a diagonal line indicates subjects known to be deceased. Black filled symbol, MS; gray filled, unaffected obligate carrier. Heterozygote carriers (M) and wild-type (wt) genotypes are provided. MS patients with inferred genotypes are indicated with an asterisk. Organism and RefSeq accession numbers are provided for orthologs and gene name and RefSeq accession numbers for human paralogs, which were obtained from Ensembl release 91. Evolutionarily conserved positions for nominated pathogenic variants are highlighted in black. (PDF) [file pgen.1008180.s006.pdf]

Fig. S1 - Segregation analysis and conservation for a) MASP1 p.Pro462Thr and b) RNF213 p.Arg4019Cys.

Males are represented by squares and females by circles, a diagonal line indicates subjects known to be deceased. Black filled symbol, MS; gray filled, unaffected obligate carrier. Heterozygote carriers (M) and wild-type (wt) genotypes are provided. MS patients with inferred genotypes are indicated with an asterisk. Organism and RefSeq accession numbers are provided for orthologs and gene name and RefSeq accession numbers for human paralogs, which were obtained from Ensembl release 91. Evolutionarily conserved positions for nominated pathogenic substitutions are highlighted in black.

a)

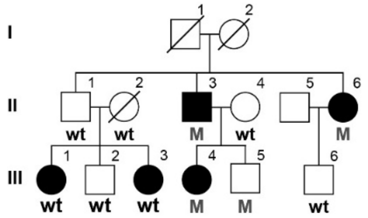


```
                                 P462T
Human NP_624302.1            AEPGLFPWQALIV
Mouse NP_032581.2            AQKGTMPWIAMLS
Rat NP_071593.1              AQKGTTPWIAMLS
Cow NP_001070436.1           AEPGLFPWQALIV
Chicken NP_998751.1          AEPGFFPWQALIV
Zebrafish XP_001341936.1     ASPGLFPWQVLLS
Frog NP_001090874.1          AEPGFFPWQVLIV

MASP1 NP_624302.1            AEPGLFPWQALIV
MASP2 NP_006601.2            AKPGDFPWQVLIL
C1R NP_001724.4              AKMGNFPWQVFTN
C1S NP_001725.1              ADIKNFPWQVFFD
C1RL NP_057630.2             AKLGNFPWQAFTS
F7 NP_062562.1               CPKGECPWQVLLL
F9 NP_001300842.1            AKPGQFPWQVVL-
F10 NP_000495.1              CKDGECPWQALLI
HP NP_005134.1               DAKGSFPWQAKMV
HPR NP_066275.3              DAKGSFPWQAKMV
PROC NP_000303.1             TRRGDSPWQVVLL
PROZ NP_001243063.1          -DLQDLPWQVKLT
```

b)

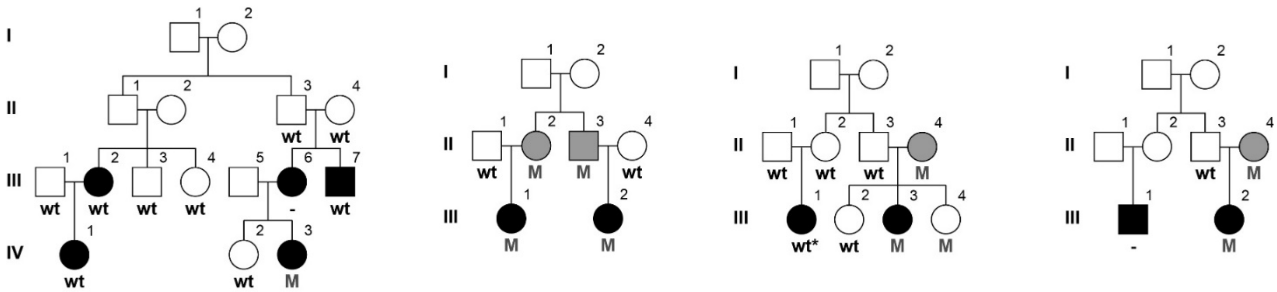

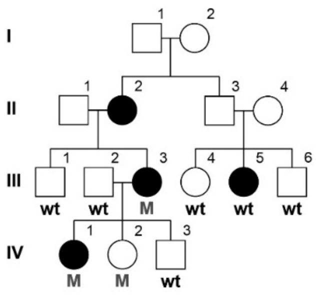


```
                                 R4019C
Human NP_001243000.2         DHVHCLRCLRAWF
Mouse XP_006534693.1         DHVYCLRCIQTWL
Rat XP_017453312.1           DHVYCLPCIQTWL
Cow XP_015323737.1           DHVFCLRCIEVNL
Chicken XP_004946335.2       NHAFCEKCIRAWL
Zebrafish NP_001340764.1     DHIYCLTCIRQWL
Frog XP_017945222.1          -------------
```
