# Supplementary material for: Exome sequencing in multiple sclerosis families identifies 12 candidate genes and nominates biological pathways for the genesis of disease
Source: PLoS Genet. 2019 Jun 6;15(6):e1008180. doi: 10.1371/journal.pgen.1008180 (PMC6553700; doi:10.1371/journal.pgen.1008180)

Fig. S2 - NLRP12 inhibition of NF-κB pathways.

Relative NF-κB promoter activity ± standard error for wild-type and mutant NLRP12 constructs is provided; n.s., not significant.

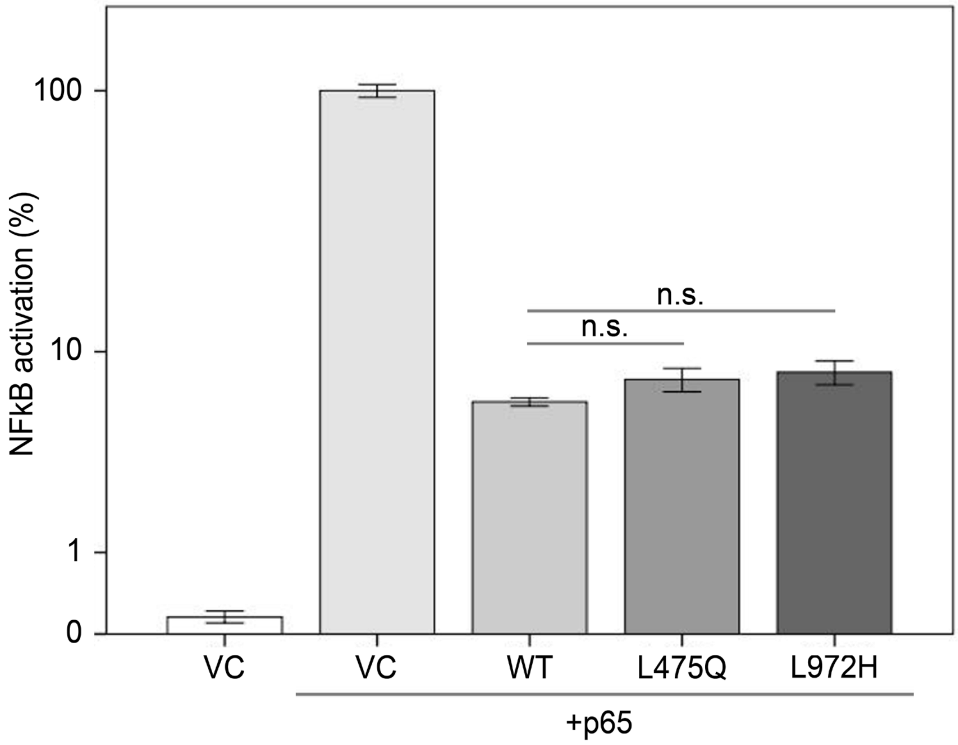

Supplement: S2 Fig — Relative NF-κB promoter activity ± standard error for wild-type and mutant NLRP12 constructs is provided; n.s., not significant. (PDF) [file pgen.1008180.s007.pdf]
